# Association between Obstructive Sleep Apnea and Heart Failure in Adults—A Systematic Review

**DOI:** 10.3390/jcm12196139

**Published:** 2023-09-22

**Authors:** Agnieszka Polecka, Natalia Olszewska, Łukasz Danielski, Ewa Olszewska

**Affiliations:** 1 Doctoral School of the Medical University of Bialystok, 15-089 Bialystok, Poland; 2 Student Research Group, Department of Otolaryngology, Medical University of Bialystok, 15-089 Bialystok, Poland; 3 Sleep Apnea Surgery Center, Department of Otolaryngology, Medical University of Bialystok, 15-089 Bialystok, Poland

**Keywords:** sleep, sleep apnea, obstructive sleep apnea, OSA, sleep-disordered breathing, heart failure

## Abstract

Background: Heart failure (HF) patients commonly experience obstructive sleep apnea (OSA), which may worsen their condition. We reviewed a diverse range of studies to investigate the prevalence of OSA in HF patients, the effects of positive airway pressure (PAP) treatment, and the potential impact of sodium-glucose cotransporter-2 inhibitors (SGLT2i) and sacubitril/valsartan on OSA outcomes. Methods: We analyzed case-control, observational studies, and randomized controlled trials. Prevalence rates, PAP treatment, and HF pharmacotherapy were assessed. Results: Numerous studies revealed a high prevalence of OSA in HF patients, particularly with preserved ejection fraction. PAP treatment consistently improved an apnea-hypopnea index, left ventricular ejection fraction, oxygen saturation, and overall quality of life. Emerging evidence suggests that SGLT2i and sacubitril/valsartan might influence OSA outcomes through weight loss, improved metabolic profiles, and potential direct effects on upper airway muscles. Conclusions: The complex interplay between OSA and HF necessitates a multifaceted approach. PAP treatment has shown promising results in improving OSA symptoms and HF parameters. Additionally, recent investigations into the effects of HF pharmacotherapy on OSA suggest their potential as adjunctive therapy. This review provides insights for clinicians and researchers, highlighting the importance of addressing OSA and HF in patient management strategies.

## 1. Introduction

Obstructive Sleep Apnea Syndrome (OSAS) is a chronic inflammatory disease characterized by episodes of total or partial obstruction of upper respiratory airways during sleep with preserved respiratory muscle effort [1]. In accordance with the American Academy of Sleep Medicine Task Force definition, obstructive sleep apnea (OSA) is characterized by the occurrence of five or more respiratory events per hour of sleep, which is measured by the apnea-hypopnea index (AHI) [2]. Clinically, OSA may manifest with the following symptoms: daytime sleepiness, loud snoring, arousals caused by gasping or choking, concentration and memory impairment, morning headaches, mood disorders, or insomnia. Moreover, the sleep partner of the patient may observe their apneas, gasping, or choking [2]. The severity of obstructive sleep apnea is classified as mild (AHI = 5–14), moderate (AHI = 15–29), or severe (AHI ≥ 30) [3]. The prevalence of OSA is estimated at 44% in the general European adult population, with approximately 23% of patients with moderate to severe OSA (AHI ≥ 15) [4]. Unfortunately, a significant number of individuals with OSA remain undiagnosed or untreated. Such patients are predisposed to an elevated risk of hypertension, cardiovascular disease (CVD), heart failure, stroke, metabolic derangements (obesity, diabetes mellitus), depression, excessive daytime sleepiness that may lead to traffic, and work-related accidents as well as absence at work [5,6,7,8]. The pathogenesis of OSA is multifactorial and remains only partially explained. It encompasses various mechanisms, including selective activation of inflammatory pathways, endothelial dysfunction, metabolic dysregulation, and oxidative stress [9,10,11]. Endothelial dysfunction is considered one of the earliest identifiable and potentially reversible abnormalities during the progression of atherosclerosis [12]. American Academy of Sleep Medicine (AASM) offers evidence-based recommendations for the diagnosis, management, and long-term care of patients with OSA [2].

Heart failure (HF) is characterized by structural and/or functional impairments in cardiac ejection, leading to a complex clinical syndrome with distinctive symptoms and manifestations. HF has been identified as a global pandemic, with an estimated 64.3 million individuals worldwide in 2017 [13]. The prevalence of HF is expected to increase due to enhanced survival rates following an HF diagnosis. That is attributed to the availability of HF evidence-based treatment methods and the overall extended life expectancy of the general population. According to the classification based on left ventricular ejection fraction (LVEF), heart failure was categorized into three groups: HF with reduced ejection fraction (HFrEF), HF with mildly reduced ejection fraction (HFmrEF), and HF with preserved ejection fraction (HFpEF). These categories were defined based on LVEF ranges of ≤40%, 41–49%, and ≥50%, respectively. Evidence-based recommendations for the diagnosis and management of heart failure are found in the 2022 guidelines of the American College of Cardiology/American Heart Association/Heart Failure Society of America (ACC/AHA/HFSA) and the 2021 guidelines of the European Society of Cardiology (ESC) [14,15].

OSA is highly associated with adverse outcomes in heart failure patients. It possesses a potential negative feedback loop and worsens comorbid conditions that deteriorate OSA. HF and OSA complications create a vicious circle of reciprocal correlations [16]. Among patients with symptomatic or decompensated HF, the prevalence of sleep apnea ranges up to 80%. More than half of these individuals suffer from OSA [16,17]. Sleep apnea, whether in the presence or absence of HF, is associated with a higher risk of negative cardiovascular outcomes, including aggravation of HF-related symptoms, increased hospitalizations, and higher mortality rates.

Moreover, individuals diagnosed with OSA (without a previous diagnosis of HF) meet a notably elevated risk of developing HF [18]. This association between OSA and HF is influenced by various pathophysiological mechanisms, including the activation of neurohormonal pathways, increased levels of oxidative stress and inflammation, acute changes in preload and afterload due to significant swings in intrathoracic pressure, and the exacerbation of systemic hypertension. An incident of airflow obstruction, hypoxia, and an attempted inspiratory effort result in arousal and an exaggerated drop in intrathoracic pressure. The drop in intrathoracic pressure leads to the pressure increase within the left ventricle (LV), known as transmural pressure, which subsequently raises the afterload. Additionally, intrathoracic pressure drop increases the venous return, leading to distention of the right ventricle (RV) and a leftward shift of the interventricular septum.

**Figure 1:**
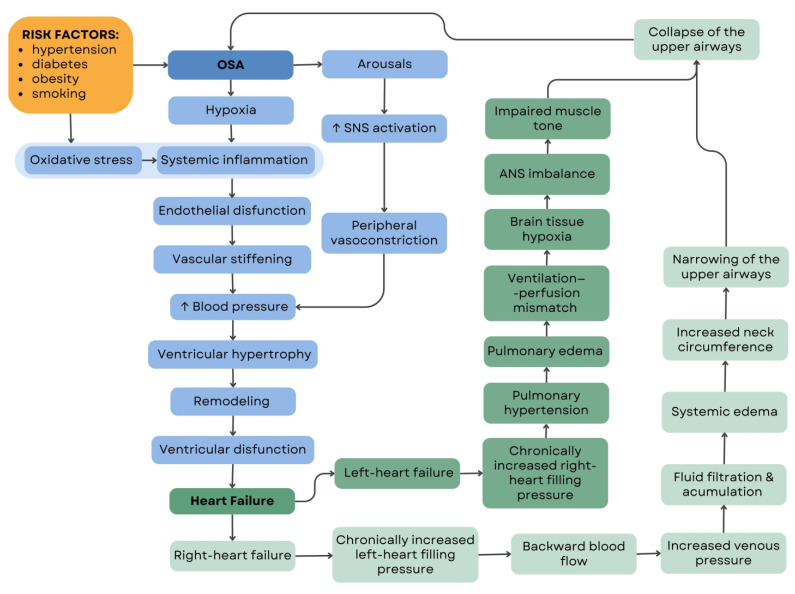
Pathophysiological association of heart failure and obstructive sleep apnea.

Consequently, a decrease in LV filling is observed. The combination of reduced LV filling and increased afterload results in a reduction in stroke volume (SV). The enlargement of the jugular vein observed in individuals with decompensated HF may significantly deteriorate OSA symptoms by exerting additional pressure on the hypopharynx, particularly in a supine position. The pathophysiological cycle showing the association between heart failure and obstructive sleep apnea is presented in Figure 1.

The primary objective of this systematic review is to comprehensively analyze the existing literature concerning the intricate interplay between OSA and HF (OSA + HF). By conducting this review, the authors aim to shed light on the mutual impacts of these two conditions, explaining how OSA influences the progression and outcomes of HF and vice versa. This endeavor is important as it enhances our understanding of the complex relationship between OSA and HF, ultimately contributing to improved patient management and healthcare strategies. One of the novel aspects this review brings is the exploration of the effect of emerging HF pharmacotherapies, specifically sodium-glucose cotransporter-2 inhibitors (SGLT2i) and sacubitril/valsartan (S/V), on sleep parameters in OSA + HF patients. The exploration of the impact of these HF pharmacotherapies in the treatment of HF + OSA significantly contributes to the existing body of knowledge in the following ways. Firstly, studying the effects of emerging HF medications on sleep parameters in the context of OSA addresses a complex dual health challenge many patients face. Secondly, the study aligns with the growing emphasis on holistic patient care. It acknowledges that HF + OSA patients require comprehensive treatment addressing cardiovascular health and sleep quality.

Additionally, the use of emerging HF pharmacotherapies reduces the number of hospitalizations, improves cardiac outcomes, and enhances the life quality of HF patients. Investigating how evolving HF medications impact sleep parameters may uncover synergistic benefits, enhancing the overall well-being of the patients. This correlation may help clinicians make more conscious decisions about treatment combinations, considering the cardiovascular and sleep-related aspects of care.

Moreover, it may indirectly contribute to better adherence to the prescribed treatment regimen, improving clinical outcomes. Studying these effects can contribute to the development of personalized treatment plans and adjusting medication to the individual needs of the patient. With these innovative medications revolutionizing HF management by targeting underlying pathophysiological pathways, it is imperative to elucidate whether they influence sleep characteristics in affected individuals. HF medications may indirectly impact sleep patterns. Therefore, understanding any potential changes in sleep parameters holds great clinical significance.

## 2. Materials and Methods

The criteria of the Preferred Reporting Items for Systematic Reviews and Meta-Analysis (PRISMA) checklist were followed in conducting and reporting this systematic review [19]. The study protocol was not registered. The PICO (population, indicator, control, outcome) questions are shown in Table 1.

We searched PubMed, Scopus Library and Cochrane for case-control studies, randomized control trials (RCTs) and observational studies concerning the prevalence of obstructive sleep apnea syndrome in heart failure patients, changes in sleep and cardiological parameters after PAP therapy, and the role of new cardiac pharmacotherapy in OSA + HF patients. The search was performed using the words “sleep apnea”, “disordered breathing”, “heart failure”, “preserved ejection fraction”, “mildly reduced ejection fraction” and “reduced ejection fraction” in different combinations.

We searched the PubMed database using the following string: ((sleep apnea) OR (OSA) OR (disordered breathing)) AND (heart failure)) and ((sleep apnea) OR (OSA) OR (disordered breathing) AND (sglt2i) OR (dapagliflozin) OR (empagliflozin) OR (ertugliflozin) OR (canagliflozin) and (sleep apnea) AND (sacubitril/valsartan)). Filters: Randomized Control Trials.

To obtain literature from the Scopus library, we used the following string: TITLE-ABS-KEY ((sleep AND apnea OR obstructive AND sleep AND apnea OR sleep AND disordered AND breathing AND heart AND failure AND (LIMIT-TO (OA, “all”) OR LIMIT-TO (OA,”Randomized Control Trials”)) AND (LIMIT-TO (PUBSTAGE, “final”)) AND (LIMIT-TO (SUBJAREA, “MEDI”)) AND (LIMIT-TO (DOCTYPE, “ar”)) AND (LIMIT-TO (LANGUAGE, “English”)) AND (LIMIT-TO (EXACTKEYWORD, “Human”)) and ((sleep apnea OR obstructive sleep apnea OR sleep disordered breathing AND empagliflozin OR dapagliflozin OR canagliflozin OR ertugliflozin OR sotagliflozin OR sacubitril valsartan AND (LIMIT-TO (OA,”all”) OR LIMIT-TO (OA,”Randomized Control Trials”)) AND (LIMIT-TO (PUBSTAGE,”final”)) AND (LIMIT-TO (SUBJAREA,”MEDI”)) AND (LIMIT-TO (DOCTYPE,”ar”)) AND (LIMIT-TO (LANGUAGE,”English”)) AND (LIMIT-TO (EXACTKEYWORD,”Human”)).

We searched Cochrane using the following string: “OSA” and “heart failure”, “heart failure” and “SDB”, “heart failure” and “sleep apnea”, “OSA” and “dapagliflozin”, “OSA” and “empagliflozin”, “OSA” and “canagliflozin”, “OSA and ertugliflozin”, and “OSA and sotagliflozin”.

The search results were exported to the Mendeley reference manager for the records’ initial title and abstract screening. Duplicate articles were removed by the “remove duplicates” function of Mendeley. The literature search was performed between 2 June 2023 and 20 June 2023 and again on 2 July 2023. To obtain articles that were not received from databases, bibliographies of published articles were manually reviewed to identify additional studies. Two authors (A.P. and N.O.) independently performed the literature search and evaluated articles for inclusion. Discrepancies, if any, were resolved through discussion.

During the initial screening of titles and abstracts, the retrieved studies had to meet the following criteria for inclusion in full-text eligibility assessment: (1) randomized control trials, case-control studies or observational studies; (2) papers concerning adult human subjects with HF; (3) papers concerning adult human subjects diagnosed with OSA (AHI ≥ 5), (4) studies evaluating a combination of sleep and/or cardiological parameters, (5) clearly defined experimental and control groups. Exclusion criteria were: (1) studies in other than English language, (2) studies on pediatric population (i.e., age < 18 years), (3) the studies were classified as article review, letter, poster, conference summary or editorial, (4) the studies were not a randomized control trial/case-control/observational study. After the initial screening, two investigators (A.P. and N.O.) retrieved and independently assessed full-text manuscripts. 

**Figure 2:**
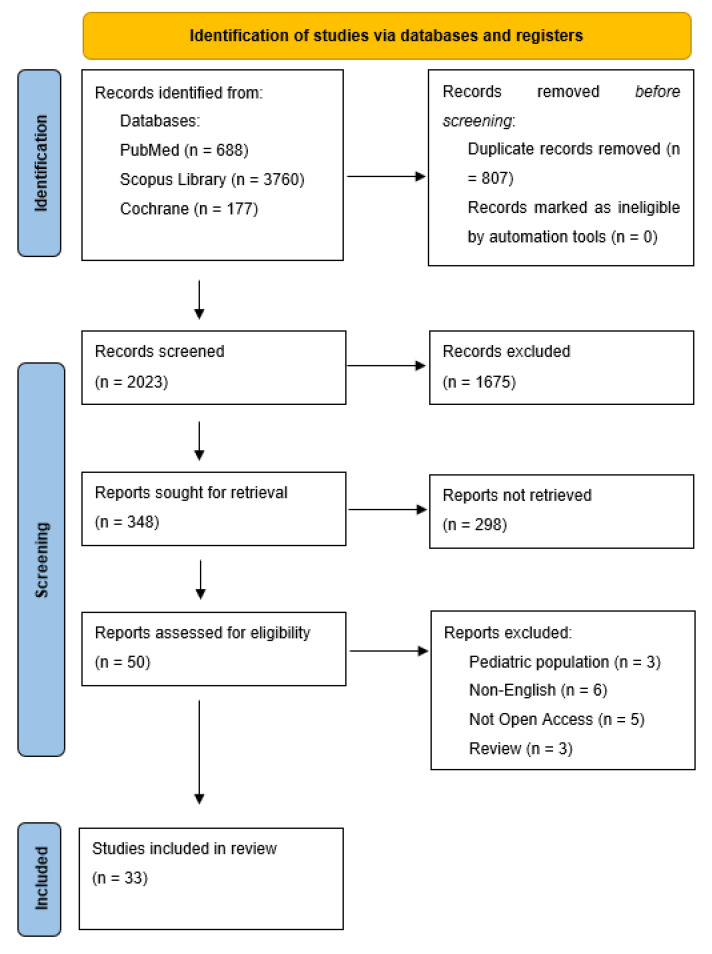
PRISMA flow diagram of the literature selection process.

The process for selecting the studies is provided in the flow chart in Figure 2.

The Quality Assessment Tool EPHPP (Effective Public Healthcare Panacea Project) was used to evaluate the quality of the studies included in our systematic review. Two authors (A.P. and N.O.) performed an independent search and evaluation of the studies following the Quality Assessment Tool for Quantitative Studies Dictionary. Any discrepancies or concerns that arose during this process were thoroughly discussed by the authors to ensure consistency and accuracy in the evaluation process. This tool enabled us to thoroughly assess the quality of various study types (e.g., randomized controlled trial, controlled clinical trial, cohort, case-control) by offering customizable criteria based on study design. It facilitated a comprehensive evaluation of study quality by examining selection bias, study design, data collection methods, blindings, and potential confounding variables. Quality assessment of included studies is presented in Supplementary Material, Table S1. Comprehensive information on the assessment process and the specific questions used for evaluation are presented in Supplementary Material, Table S2.

## 3. Results

### 3.1. The Prevalence of Obstructive Sleep Apnea in the Heart Failure Population

The prevalence of OSA in HF patients was estimated by Wang et al. and depended on the LVEF [20]. Among 252 HF patients enrolled in the study, 48% presented OSA as well. When comparing the HFrEF, HFmrEF, and HFpEF groups, there were 42%, 47%, and 49% of OSA participants, respectively (*p*  = 0.708). Additionally, the prevalence and the severity of sleep-disordered breathing (SDB) were significantly higher in HFrEF and HFmrEF. The above-mentioned types of heart failure were associated with central sleep apnea (CSA). OSA was found to be more common in individuals with HFpEF. 

Wang et al. conducted another study with 248 patients diagnosed with heart failure to explore the prevalence of sleep-disordered breathing in patients with HF of different etiologies. The overall prevalence of SDB in the HF population was 70.6%, with OSA accounting for 47.6%. The patients were categorized into five groups based on the underlying cause of HF: ischemic, hypertensive, myocardial, valvular, and arrhythmic. The prevalence of SDB across these five groups was 75.3%, 81.4%, 77.8%, 51.9%, and 58.5%, respectively (*p* = 0.014). Regarding OSA, the prevalence among the five groups was 42.7%, 72.1%, 36.1%, 37.0%, and 49.1%, respectively (*p* = 0.009) [21]. An analysis of sleep data across the five groups revealed that AHI, the longest duration of hypopnea, and the proportion of Cheyne-Stokes respiration (CSR) were higher in the ischemic, hypertensive, and myocardial groups compared to the valvular and arrhythmic groups (18.3 (5.0–31.4); 12.8 (6.1–28.0); 20.3 (9.3–34.5), respectively; *p* < 0.05). The myocardial group had the lowest LVEF values, followed by the ischemic group, whereas the other three groups demonstrated higher LVEF values (0.43 (0.31–0.52); 0.58 (0.43–0.68), respectively; *p* < 0.001).

Gupta et al. screened two groups of patients for SDB: 25 individuals previously diagnosed with HFpEF and 25 age and sex-matched controls of healthy subjects. SDB was observed in 64% of the case patients and 12% of the control group (*p* < 0.001). Among HFpEF patients with SDB (16/25), 13 were diagnosed with OSA and 3 with CSA. There was a significant difference between the patients and controls in AHI (*p* < 0.001), NT-ProBNP (*p* < 0.001), and polysomnography parameters (PSG WASO, PSG N1, N2, N3). A positive correlation between the AHI score and the degree of diastolic dysfunction was observed (r  =  0.67; *p* < 0.001) [22].

The German multicenter SchlaHF (Sleep-Disordered Breathing in Heart Failure) registry by Arzt et al. enrolled 1557 HFrEF patients and estimated OSA as 29% of all included individuals [23]. 

Oldenburg et al. screened 700 patients with HF for SDB and presented 76% of SDB in the studied population, including 36% of OSA [16]. Patients with no SDB (including OSA) experienced less severe symptoms (New York Heart Association (NYHA) class 2.57 ± 0.5; *p* < 0.05) compared to the individuals with CSA (NYHA class 2.9 ± 0.5). Additionally, OSA patients had significantly higher LVEF values (*p* < 0.05) than CSA patients.

Bitter et al. investigated the prevalence and type of SDB in patients with HFpEF. The authors enrolled 244 patients with HFpEF and documented SDB in 169 patients (69.3%), of which 97 (39.8%) had OSA. The severity of OSA was mild in 40%, moderate in 36%, and severe in 24% of the cases [24].

A study conducted by Chan et al. screened 20 patients with HFpEF for SDB. 55% of participants were diagnosed with significant sleep-disordered breathing. In this group, 63.64% of patients had predominantly OSA with a mean AHI of 10.9 ± 5.1 [25].

Yumino et al. enrolled 218 patients with HF (with LVEF ≤ 45%) and screened them for SDB. Using AHI cutoff ≥ 10, ≥15, and ≥20, the prevalence of sleep apnea was estimated as 60%, 47%, and 39%, respectively. The prevalence of OSA was 37%, 26%, and 21%, respectively. The results of the OSA population were BMI (31.0 ± 5.0), LVEF (25.7 ± 9.1), and NYHA Class (class III + IV: OSA 31) [26]. 

Herrscher et al. assessed the prevalence of SDB in HF patients independent of systolic left ventricular function. In a cohort of 115 patients (62% with reduced EF and 38% with preserved EF), individuals were classified as New York Heart Association Class II–IV. The prevalence of SDB was 81%, including 54% of OSA. Among the HFpEF patients, SDB was present in 80% of the cases, with OSA occurring in 62%. Furthermore, the group of HFpEF patients also revealed a significantly higher incidence of hypertension. When comparing patients with preserved EF to the ones with reduced EF, both groups had nearly the same high prevalence of sleep apnea (80% vs. 82%). Additionally, within the HFpEF group, there were more patients with OSA than CSA (62% vs. 18%) [27].

Kalaydzhiev et al. screened 100 individuals and found 61 sleep-disordered breathing patients. In this study population, 50 individuals were diagnosed with OSA (82%), and 52% were male. The following parameters were estimated: left ventricular ejection fraction at 49.6 ± 8.5%, AHI at 41.8 ± 23.2, BMI at 38.5 ± 7.1, NTproBNP at 1359.12 ± 740.64 pg/mL, mean oxygen saturation (MOS) at 83.9 ± 6.8%, and the lowest oxygen saturation (LOS) at 65.3 ± 12.7% [28].

**Figure 3:**
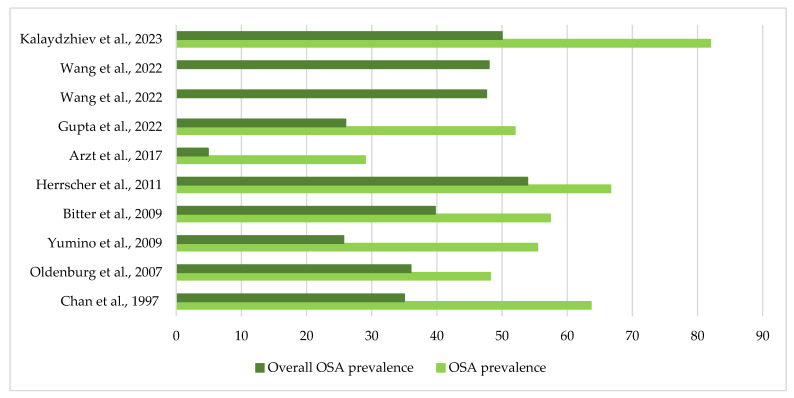
OSA prevalence in chosen studies. Abbreviations: Overall OSA prevalence, number (n) of OSA/n all study participants; OSA prevalence, n OSA/n SDB participants [16,20,21,22,23,24,25,26,27,28].

The summarized data of chosen studies is presented in Table 2. Figure 3 presents OSA prevalence over the years.

### 3.2. Does Positive Airway Pressure Play a Role in HF Patients?

#### 3.2.1. Obstructive Sleep Apnea and Heart Failure with Preserved Ejection Fraction

Arikawa et al. collected data from 58 patients with new-onset HFpEF. In these patients, LVEF and plasma BNP concentration at the baseline were 61 ± 5% and 391 (218–752) pg/mL, respectively. Obstructive sleep apnea, with a mean AHI score of 43 ± 16, was found in 39 subjects (67%). Furthermore, none of these patients showed evidence of CSA. All of them were treated for OSA with CPAP and were advised lifestyle modifications over a 36-month observational period. The baseline plasma brain natriuretic peptide concentration in the studied groups was 444 (233–752) pg/mL in OSA and 316 (218–703) pg/mL in the non-OSA group. After 36 months of the follow-up period, the BNP concentration decreased in both groups. However, the reduction was less significant in patients with sleep apnea. While the BNP concentration was similar at the one-month cutoff in both groups, they were notably higher in the sleep apnea group after six months (*p* < 0.05), 12 months (*p* < 0.05), and 36 months (*p* < 0.05) [29]. This research demonstrated that in patients with HFpEF, obstructive sleep apnea leads to elevated BNP levels during extended follow-up periods compared to non-OSA subjects. These findings indicate that even with appropriate CPAP treatment, OSA might negatively impact long-term cardiac function and prognosis. Table 3 presents data from the study.

#### 3.2.2. Obstructive Sleep Apnea and Heart Failure with Reduced Ejection Fraction

The prospective, single-arm, open-label study conducted by Naito et al. analyzed 55 Japanese patients with HFrEF and moderate-to-severe OSA [30]. After one month of CPAP treatment, the AHI decreased from 45.3 ± 16.1 to 5.4 ± 4.1 and the arousal index from 43.9 ± 19.6 to 15.7 ± 10.3. The LVEF improved from 37.2% ± 9.8 to 43.2% ± 11.7. Additionally, a significant decrease in heart rate (76.3 ± 11.2 vs. 70.7 ± 9.0, *p* < 0.001), systolic (131.3 ± 13.3 vs. 126.2 ± 12.2, *p* < 0.001), and diastolic (78.4 ± 10.5 vs. 74.3 ± 10.3, *p* < 0.001) blood pressure after the treatment was noted. However, there were no significant changes in the BMI. Univariate regression analysis showed that age (*p* < 0.001), BMI (*p* < 0.001), atrial fibrillation (*p* = 0.0443), LOS (*p* = 0.0266), and pressure levels of CPAP (*p* = 0.0013) were positively associated with improvements in LVEF at the baseline. After adjusting for confounding variables, age (*p* = 0.008) and BMI (*p* < 0.001) became the most significant factors for LVEF improvement. There was no correlation between pharmacotherapy (ACE inhibitors, AR blockers, Beta-blockers, diuretics, spironolactone, nitrates, digoxin) and LVEF improvement in HFrEF patients. The multivariate regression analyses indicated that young patients with obesity are inclined to LVEF enhancement. The degree of improvement was estimated at 6% in this population. The results are consistent with other studies. 

Kaneko et al. conducted a study on 24 patients previously diagnosed with OSA and HFrEF [31]. The subjects were randomly assigned to control (N = 12) and CPAP group (N = 12). After one month, a significant reduction in the following sleep parameters was observed in the CPAP group: AHI (from 31.7 ± 6.4 to 8.3 ± 2.8, *p* < 0.001), arousal index (from 31.4 ± 6.1 to 12.8 ± 1.7, *p* = 0.003) and desaturation index (from 12.7 ± 3.2 to 0.8 ± 0.5, *p* < 0.001). Moreover, the LOS improved from 82.3 ± 1.2 to 89.6 ± 1.1 (*p* = 0.004). Additionally, there was a decrease in cardiological parameters, such as: daytime systolic blood pressure (from 126 ± 6 to 116 ± 5 mmHg, *p* = 0.02) and heart rate (from 68 ± 3 to 64 ± 3 beats per minute, *p* = 0.007). Furthermore, the LVEF value increased from 25.0 ± 2.8% to 33.8 ± 2.4%. There were no significant improvements in the control group in the above-mentioned parameters. In conclusion, patients who received HF treatment and managed concurrent OSA through CPAP presented systolic blood pressure and HR reduction, as well as left ventricular systolic function improvement.

Mansfield et al. enrolled 55 individuals formerly diagnosed with both OSA and HFrEF. The study was randomized: 28 subjects were assigned to the CPAP group and 27—to the control group. The results indicated that three months of CPAP treatment was associated with significant improvements in LVEF (Δ1.5 ± 1.4% vs. 5.0 ± 1.0%, respectively, *p* = 0.04), AHI (Δ−8.4 ± 3.6 vs. −21.1 ± 3.8, *p* < 0.001), LOS (Δ0.0 ± 1.6 vs. 11.5 ± 2.7, *p* = 0.001), reductions in overnight urinary norepinephrine excretion (Δ1.6 ± 3.7 vs. −9.9 ± 3.6 nmol/mmol creatinine, *p* = 0.036), and improvements in quality of life (in the domains of physical role (*p* = 0.03), vitality (*p* = 0.02), social functioning (*p* = 0.03), and mental health (*p* = 0.01). Overall, the treatment of OSA among HF patients leads to improvement in cardiac function, sympathetic activity, and quality of life [32]. 

Fox et al. randomized 58 patients with HFrEF and OSA to automatic positive airway pressure (Auto-PAP) (N = 25) or nasal strips (controls) (N = 33) [33]. The study indicated significant LVEF improvement in the Auto-PAP group (from 38 ± 9% at baseline to 40 ± 9% at six months) compared with controls (40 ± 9% to 40 ± 8%; *p* < 0.01). AHI decreased significantly from baseline to 6 months in the Auto-PAP group (from 34 ± 17/h to 9 ± 8/h; *p* < 0.001) but remained unchanged in the control group (from 35 ± 13/h to 33 ± 20/h). Additionally, patients with Auto-PAP treatment experienced a greater improvement in the MOS (controls: from 92.03 ± 2.23 to 92.00 ± 2.97, *p* = 0.857; Auto-PAP group: from 92.47 ± 2.62 to 93.82 ± 1.92, *p* = 0.001) when compared to both initial levels and the control group. In summary, Auto-PAP intervention demonstrated a significant improvement compared to the control group, especially in terms of percent-predicted cardiopulmonary exercise capacity (peak VO2), a well-established marker for cardiovascular prognosis in HFrEF. Additionally, Auto-PAP showed beneficial effects on hypoxemia, cardiac function, and overall quality of life.

A randomized sham-controlled trial conducted by Kim et al. on 52 patients with severe OSA and reduced ejection fraction analyzed left ventricle (LV) and right ventricle (RV) function by conventional and speckle-tracking echocardiography before and after three months of CPAP (N = 26) or sham treatment (N = 26) [34]. CPAP treatment significantly improved LV global longitudinal strain (GLS) compared to the sham treatment (−20.0% ± 2.1% vs. −18.0% ± 2.5%; *p* = 0.004). There were no differences in LV dimension or ejection fraction. CPAP treatment reduced RV size and improved the fractional area change (51.3% ± 7.9% vs. 46.9% ± 6.7%; *p* = 0.038) compared with the sham treatment but did not improve the RV GLS compared with the sham treatment. Overall, in individuals diagnosed with severe OSA, three months of CPAP therapy resulted in enhancement of LV and RV function when compared to the sham treatment. CPAP treatment significantly improved, especially in LV mechanical function and RV fractional area change evaluated through speckle-tracking and two-dimensional echocardiography.

Gilman et al. performed a substudy of a larger randomized controlled trial on 19 OSA and HFrEF individuals and randomized them to CPAP treatment (N = 12) or control (N = 7) group for one month [35]. In the control group, there were no significant changes in the AHI (from 41 ± 13 to 37 ± 18) and other sleep parameters (LOS) from 82.4 ± 6.9 to 78.5 ± 12.4; arousals from 34.1 ± 13.5 to 35.1 ± 15.8) between the baseline and the follow-up. However, in contrast, CPAP intervention, with an average pressure of 8.8 ± 2.4 cm H_2_O and a nightly usage duration of 6.3 ± 1.5 h, resulted in significant reductions in AHI (from 30 ± 15 to 7 ± 6), arousal index (from 26.7 ± 10.3 to 11.6 ± 3.6) (all *p* < 0.001). Furthermore, CPAP therapy led to improvements in MOS (from 94.8 ± 1.0 to 96.1 ± 1.6, *p* = 0.022) and LOS (from 82.5 ± 5.1 to 90.5 ± 3.6, *p* < 0.001). In the control group, there was no significant change in LVEF between baseline and follow-up. In contrast, the CPAP group presented an increase in LVEF, significantly greater than the control group (*p* = 0.028).

Servantes et al. conducted a study to examine the effects of exercise training and CPAP in patients with HFrEF (LVEF < 40%) and OSA. A total of sixty-five participants enrolled in four groups: control group (N = 18), exercise group (N = 17), CPAP group (N = 15), and exercise + CPAP group (N = 15), completed the study protocol. CPAP adherence and average daily use were similar between the groups. When comparing the baseline measurements with the three-month follow-up, there was no significant change in the mean AHI in the control group. However, the exercise group demonstrated a moderate decrease in AHI from 28 ± 17 to 18 ± 12 (*p* < 0.03). In contrast, both the CPAP group and the exercise + CPAP group exhibited a significant reduction in AHI, from 32 ± 25 to 8 ± 11 (*p* < 0.007) and from 25 ± 15 to 10 ± 16 (*p* < 0.007), respectively. No significant changes were observed in NYHA functional class distribution, excessive daytime sleepiness, quality of life, or sexual function in the control group. However, in the other intervention groups, there was an improvement in the NYHA functional class (classes II and III moved into class I, *p* < 0.05) and a reduction in daytime sleepiness (*p* < 0.05). Significant improvements in quality of life were observed in the exercise and exercise + CPAP groups compared to the control group (*p* < 0.05). Sexual function improved in the exercise + CPAP group compared to baseline, with no significant differences among the groups [36]. 

Egea et al. selected 60 patients with HF with LVEF < 45% and sleep apnea (83% OSA, 17% CSA) with AHI > 10/h and evaluated them at baseline and after three months of treatment with optimal CPAP or sham-CPAP. An improvement in AHI and LVEF was observed in the CPAP group but not in the sham group. In patients with HF and OSA, there was an improvement in the LVEF in the patients treated with CPAP but no changes in the sham-CPAP group after three months of treatment (*p* = 0.03) [37]. 

Ryan et al. enrolled 18 patients with OSA and HF with LVEF < 45% and randomized them to the control group (N = 8) and the CPAP group (N = 10). Over one month, there were no changes in participants’ BMI, diastolic blood pressure, or heart rate. After one month, a significant reduction in AHI was observed in the CPAP group (*p* < 0.001), with no improvement in controls (*p* = 0.77). The improvement in LVEF was observed in the CPAP group (*p* = 0.03), and no improvement in controls (*p* = 0.18). Additionally, in the CPAP group, there was a reduction in arousal index (*p* = 0.004), ventricular premature beats (VPBs) (*p* = 0.037), and an increase in minimum SaO2 (*p* = 0.05) [38]. 

Table 4 summarizes data from the above-mentioned studies.

### 3.3. Healthcare Resources Utilization in Heart Failure and Obstructive Sleep Apnea Patients

Cistulli et al. conducted a retrospective observational study of 4237 patients with HFpEF who received a new diagnosis of OSA. The adherence to Positive Airway Pressure (PAP) therapy was associated with improvements in healthcare resource use, including reductions in general hospitalization rate (adherent to PAP 0.33 ± 0.84 vs. nonadherent 0.53 ± 1.08, *p* < 0.001), cardiovascular hospitalizations (adherent to PAP 0.06 ± 0.28 vs. nonadherent 0.11 ± 0.41, *p* < 0.004.), and emergency room visits (adherent to PAP 0.83 ± 1.49 vs. nonadherent 1.21 ± 1.82, *p* < 0.001). The study observed the clinical and economic benefits of OSA treatment in patients with heart failure with preserved ejection fraction, particularly among individual’s adherent to therapy. Adherent patients had lower total healthcare costs than moderately adherent and nonadherent patients, with $12,676 vs. $16,157 vs. $16,173, respectively (*p* < 0.001 for both comparisons). Furthermore, adherent patients had significantly reduced costs associated with inpatient hospitalizations ($3880 vs. $6409, vs. $7025, respectively; *p* < 0.001 for both comparisons) and emergency room visits ($741 vs. $1142, vs. $1168, respectively; *p* < 0.001 for both comparisons) [39]. 

The correlation between OSA and the risk of hospitalization in patients with HF was analyzed by Abdullah et al. The researchers examined data from the National Inpatient Sample (NIS), Healthcare Cost and Utilization Project, and Agency for Healthcare Research and Quality database, specifically focusing on records between 2012 and 2014. A total of 12,608,637 hospital discharges of adult patients were included in the analysis. Among the data, there were 147,463 patients with a primary diagnosis of HFpEF. There were 653,762 (5.2%) patients with OSA. The prevalence of OSA in patients with HFpEF and without HFpEF was estimated at 16.8% and 5.0%, respectively. Patients with OSA were older (62.5 ± 13.7 vs. 58.6 ± 20.8, *p* < 0.001) and predominantly male, smoked (35.4% vs. 28.0%, *p* < 0.001) and had a higher incidence of comorbidities, including hypertension (78.1% vs. 53.9%, *p* < 0.001), coronary artery disease (35.3% vs. 20.7%, *p* < 0.001), prior myocardial infarction (8.9% vs. 5.2%, *p* < 0.001), atrial fibrillation (26.1% vs. 14.1%, *p* < 0.001), chronic kidney disease (28.1% vs. 15.5%, *p* < 0.001), acute kidney injury (18.7% vs. 12.3%, *p* < 0.001), diabetes mellitus (52.8% vs. 25.9%, *p* < 0.001), obesity with BMI = 30–40 (18.7% vs. 6.0%, *p* < 0.001), and morbid obesity with BMI > 40 (31.8% vs. 3.7%, *p* < 0.001). The primary endpoint, a discharge with HFpEF diagnosis, occurred in 3.8% of patients in the OSA group and 1.0% in the non-OSA group (*p* < 0.001). Multivariable logistic regression analysis confirmed that OSA was independently associated with higher odds of admission with HFpEF. This association remained significant in both women and men, with adjusted odds ratios of 2.3 (95% CI 2.27 to 2.36) and 2.0 (95% CI 1.98 to 2.08), respectively [40].

Malhotra et al. enrolled 3182 patients with OSA and HFrEF and assessed the impact of adherence to PAP therapy on healthcare resource utilization. During the first year of therapy, 39% of patients (N = 1252) were considered adherent to PAP therapy, 29% (N = 935) had intermediate adherence, and 31% (N = 995) were nonadherent. After one year of initiating positive airway pressure treatment, patients adherent to the treatment had a lower number of combined healthcare visits compared to nonadherent patients (0.92 ± 1.59 and 1.15 ± 1.83, respectively, *p* = 0.006). This reduction was primarily attributed to a 24% decrease in emergency room visits. Additionally, the cost of combined healthcare visits was found to be statistically lower in adherent patients ($3500) compared to nonadherent patients ($5879, *p* = 0.031) [41]. Significant predictors of adhering to PAP included older age (>55 years), presence of atrial fibrillation, and adherence to β-blocker medication. Table 5 presents data on costs and hospitalization and ER risk of OSA + HF patients.

### 3.4. Do New Medicaments in Heart Failure Pharmacotherapy Play a Role in Sleep-Disordered Breathing Patients?

#### 3.4.1. Sodium/Glucose Cotransporter-2 Inhibitors (SGLT2i)

Wojeck et al., in VERTIS CV exploratory study, evaluated the impact of ertugliflozin (5 mg, 15 mg, and control group) on the prevalence of OSA. Out of 8246 enrolled patients, 93.3% (N = 7697) had no baseline OSA (placebo N = 2561; ertugliflozin N = 5136; mean age 64.4 years; BMI 31.7 kg/m^2^; HbA1c 8.2%; 69.2% male; 88.3% White). The results were: OSA incidence rate: 1.44 per 1000 person-years for ertugliflozin vs. 2.61 per 1000 person-years for placebo, resulting in a 48% relative risk reduction (HR 0.52; 95% CI 0.28–0.96; p = 0.04). In summary, in the VERTIS CV study, the use of the SGLT2 inhibitor ertugliflozin resulted in a decreased occurrence of OSA in individuals with type 2 diabetes. [42].

In a recent post-hoc analysis of the EMPA-REG OUTCOME trial to explore the effects of empagliflozin (EMPA) on the incidence of OSA, it was found that approximately 6% of the enrolled population had OSA at baseline. Patients with OSA were more likely to have moderate to severe obesity (55.2% vs. 18.2%) and a higher prevalence of coronary artery disease (CAD). Additionally, patients with OSA had an increased risk of cardiovascular and kidney events and higher overall all-cause mortality compared to those without OSA. The analysis also indicated a trend towards greater weight loss (adjusted for baseline body weight) in patients with OSA treated with empagliflozin compared to those without OSA. Interestingly, patients treated with EMPA had a 52% lower likelihood of developing new-onset OSA than those treated with a placebo. No AHI measurements were made. The diagnosis of OSA in this study was based on patient and investigator reports rather than objective assessment using systematic polysomnography [43].

Two small prospective studies assessed dapagliflozin (DAPA) in individuals with SDB. In the first study, DAPA (5 mg/day) was administered to 30 obese diabetes type 2 (T2D) patients for 24 weeks. SDB was categorized based on the 3% oxygen desaturation index (ODI). After treatment, weight loss was 1.7 kg and 2.56 kg in mild and moderate/severe SDB groups, respectively. DAPA significantly improved 3% ODI only in the moderate/severe SDB group (baseline: 25.0 ± 3.8; end: 18.5 ± 6.1, *p* = 0.017). Notably, weight loss and neck circumference reduction did not correlate with a 3% ODI improvement. Polysomnography, AHI data, and a control group were lacking in this study [44].

In the second study, 36 OSA and T2D patients were divided into two groups: the dapagliflozin (DAPA) arm (N = 18) received 5 mg/day DAPA (increased to 10 mg after one week), and the control arm (N = 18) received 2 mg/day glimepiride (titrated up to 4 mg if needed). Both groups received metformin 850 mg twice daily for 24 weeks. DAPA resulted in significant reductions in BMI, Homeostatic Model Assessment for IR (HOMA-IR), and AHI, improved minimum SpO_2_, and decreased ESS scores compared to glimepiride (*p* < 0.05). Limitations include small sample size, short duration, and absence of neck circumference and other obesity-related data. Sulfonylurea use in the control group may have affected BMI differences [45].

A retrospective study (with no control group) conducted by Sawada et al. examined the effects of SGLT2 inhibitors on 18 T2D patients with OSA (12/18 with severe OSA) regarding weight reduction and changes in AHI. SGLT2 inhibitors were administered for a median of 21 weeks. Body weight, BMI, and AHI (from 31.9 ± 18.0 to 18.8 ± 11.5, *p* = 0.003) significantly improved after treatment. The number of participants with severe OSA decreased from 12 to 4. However, greater body weight reduction was associated with less AHI improvement in severe OSA patients. Compensatory hyperphagia and concurrent diuretic therapy were suggested as possible explanations [46].

DAHOS, a 3-month, multicentric, prospective, randomized controlled clinical study by Xie et al., is conducted to assess the changes in OSA-related indicators and the treatment of heart failure and to verify the effectiveness of dapagliflozin (10 mg) in the treatment of HFrEF with coexisted OSA. Inclusion criteria are adults with LVEF ≤  40% AHI  ≥  15. Patients will be randomized to optimized HF therapy plus a standard dose of dapagliflozin, while the controls will receive only optimized HF therapy. Participants will be evaluated at baseline and 3-month follow-up after dapagliflozin administration. The primary endpoint of the main study is the decreasing value of AHI. The secondary outcomes of the study include assessing the proportion of patients experiencing a 20% and 50% decrease in the AHI before and after dapagliflozin treatment, evaluating changes in Epworth Sleepiness Scale (ESS) scores, examining echocardiographic measures of structure and function (ejection fractions, left ventricular diameters, atrial surface, diastolic function, and filling pressures) pre- and post-dapagliflozin, analyzing serum BNP and pro-BNP concentrations before and after dapagliflozin, measuring laboratory parameters such as creatinine, potassium, sodium, hemoglobin, alanine, and aspartate transaminase levels, assessing the quality of life using the Minnesota Living with Heart Failure Questionnaire and EQ-5D-3L Questionnaire, and evaluating levels of inflammatory and oxidative stress factors (IL-6, CRP) before and after dapagliflozin [47].

The summarized data of the chosen studies is presented in Table 6. 

#### 3.4.2. Sacubitril/Valsartan

Owens et al. conducted AWAKE-HF randomized, double-blind study conducted in 23 centers in the United States. Participants with HFrEF (N = 140) were randomly allocated to receive either sacubitril/valsartan (N = 70) or enalapril (N = 70) treatment. Subjects presented with undiagnosed, untreated, moderate-to-severe sleep-disordered breathing (≥15 events/h), and nearly all had OSA (N = 1 CSA). Baseline and 8-week follow-up assessments were conducted to evaluate all endpoints. After eight weeks of treatment, the mean 4% AHI changed minimally from 16.3/h to 15.2/h in the sacubitril/valsartan group and from 16.8/h to 17.6/h in the enalapril group. Mean total sleep time decreased slightly in both treatment groups at week 8 (−14 and −11 min for sacubitril/valsartan and enalapril, respectively) [48]. 

The study conducted by Pelaia et al. in 2022 evaluated the effects of a 6-month therapy with sacubitril/valsartan on hemodynamic and metabolic parameters in patients with HFrEF and sleep apnea already under treatment with CPAP. Additionally, apnea/hypopnea occurrence and oxygen saturation were examined. The authors enrolled 132 consecutive patients with HFrEF and analyzed them at baseline and 6-month follow-up. Fifty-five patients (41.7%) were diagnosed with OSA, and 77 (58.3%) had CSA. Each participant received CPAP treatment. During a temporary CPAP interruption, the sleep parameters evaluation demonstrated significant improvements. There was a notable reduction in the overall AHI (from 26.5 ± 10.4 to 21.7 ± 8.3, *p* < 0.0001), ODI (from 18.0 ± 3.7 to 13.5 ± 4.9, *p* < 0.0001), and time spent with oxygen saturation below 90% (TC90) (from 14.1 ± 4.5% to 6.8 ± 3.9%, *p* < 0.0001). Additionally, there were significant increases in mean oxygen saturation, which improved from 91.3 ± 1.9% to 92.0 ± 2.0% (*p* < 0.0001). There were significant decreases in BMI (*p* < 0.0001) and NT-proBNP concentration (*p* < 0.0001) [49]. 

The ENTRESTO-SAS trial is a six-center, prospective, open-label, real-life cohort study by Jaffuel et al., which was conducted to evaluate the sacubitril/valsartan impact on sleep apnea in HFrEF patients [50]. The authors analyzed 118 patients at baseline and 3-month follow-up. The nocturnal ventilatory polygraphy was performed. Based on the initial results, three groups were established: G1: AHIcentral ≥ 5/h and AHIobstructive <15/h; G2: AHIobstructive ≥ 15/h regardless of the AHIcentral; and G3: AHIcentral <5/h and AHIobstructive <15/h. A significant decrease in AHI was observed in G1 + G2 patients, with a median reduction of −7.10/h (range: −16.10 to 0.40), *p* < 0.001. In G1 patients, who primarily exhibited a central pattern of irregular breathing, AHI significantly decreased from a median of 22.90 (range: 16.00–43.50)/h to 19.20 (range: 12.70–31.10)/h (*p* = 0.002). The median AHI difference was −6.60 (range: −11.70 to 0.40). For G2 patients, who predominantly had an obstructive pattern, AHI decreased from a median of 30.10 (range: 26.40–47.60) to 22.75 (range: 14.60–36.90) (statistically non-significant, *p* = 0.059). The median AHI difference was −12.40 (range: −23.60 to 0.35). Around 24.4% of patients experienced a ≥50% decrease in AHI (21.6% for G1 and 37.5% for G2). Additionally, 20% of patients had an initial AHI < 15, which increased to 37.78% at three months (24.3% for G1, *p* = 0.146; 0% for G2, *p* = 0.5). NT-proBNP concentration significantly decreased in all three groups (median change of −301.00 pg/mL for G1, *p* = 0.001; −309.00 pg/mL for OSA-G2, *p* = 0.043; and −299.50 pg/mL for G3, *p* < 0.001). Approximately 51.72% of the population showed a change of over 30% in NT-proBNP values after initiating SV, with no significant differences between groups. LVEF significantly increased in G1 and G3 (median change of 2% for G1, *p* = 0.001; median change of 2% for G3, *p* = 0.016) [50].

Wang et al. conducted a study to evaluate the effect of sacubitril-valsartan on 18 HFrEF patients. Out of the total 18 patients, 50% (9 patients) had OSA, 39% (7 patients) had CSA, and 11% (2 patients) had normal breathing. After three months of sacubitril-valsartan therapy, there was a reduction in NT-pro BNP concentration (*p* < 0.001) and an improvement in LVEF (*p* < 0.001). Portable apnea monitoring showed a significant decrease in the respiratory event index (REI) following sacubitril-valsartan treatment (*p* = 0.003). Subgroup analysis based on the type of apneas revealed that both REI and the time spent below 90% saturation decreased in patients with both OSA and CSA (all *p* < 0.05) [51].

Passino et al. enrolled 51 stable HFrEF patients and switched them from an ACE-i/ARB to sacubitril-valsartan [52]. The baseline characteristics were age 65 ± 9 years, 39 males, 45% of ischemic etiology, LVEF 28.6 ± 6%, 41%, NYHA class III. Fifteen patients had OSA (29%), and 33 had CSA (65%) at nighttime. Among patients with OSA, 4 (8%), 7 (13%), and 4 (8%) had mild (i.e., AHI ≥ 5, <15), moderate (i.e., AHI ≥ 15, < 30) and severe (i.e., AHI ≥ 30) apneas, respectively. Among those with CSA,12 (23%), 8 (16%) and 13 (26%) had mild, moderate, and severe apneas, respectively. After six months of S/V administration, cardiac parameters improved. There was a relevant decrease in NTproBNP (*p* < 0.001) and an increase in LVEF (*p* < 0.001). When assessing the effects on sleep parameters in the overall population, sacubitril-valsartan administration was associated with a significant decrease in the daytime AHI (*p* < 0.001), nighttime AHI (*p* = 0.026) and the 24-h AHI (*p* < 0.001). Within the subset of individuals with OSA, the impact of medication administration did not display any nocturnal effect (*p* > 0.05). In contrast, the utilization of sacubitril-valsartan showed a significant reduction in daytime occurrences (*p* = 0.007), primarily attributed to a decrease in hypopneas (80 events (33–128) to 23 events (10–41), *p* = 0.011), rather than apneas (1 event (0–9) to 0 events (0–3), *p* = 0.51). Table 7 presents summarized data from the above-mentioned studies.

## 4. Discussion

### 4.1. The Prevalence of Obstructive Sleep Apnea in Heart Failure Patients

Heart failure and obstructive sleep apnea are prevalent conditions that often coexist and interact, leading to increased morbidity and mortality [53,54]. A prior investigation indicated that approximately 75% of individuals suffering from HF experience SDB linked to daytime sleepiness, chronic bronchitis, peripheral edema, and dyspnea [55]. The prevalence of OSA in HF patients with systolic dysfunction varied from approximately 20% to 45% [56]. These findings highlight the importance of considering OSA as a potential comorbidity in HF patients, given its impact on disease progression and outcomes. Overall, the prevalence of obstructive sleep apnea among heart failure patients has exhibited a varied trajectory over the years. Especially OSA prevalence rates within the HF population, as demonstrated by studies conducted in 1997, 2007, and 2009 by Chan et al., Oldenburg et al. and Yumino et al., were reported as 63.64%, 48.21%, and 55.45%, with corresponding overall OSA prevalence rates of 35%, 36%, and 25.69% [16,25,26]. However, it’s essential to consider that the more recent investigation by Kalaydzhiev et al. in 2023 reported a notably higher OSA prevalence of 81.97% among HF patients, while the overall OSA prevalence remained at 50% [28]. It’s worth highlighting that the study by Kalaydzhiev et al. had a sample size of 100, which could potentially introduce a risk of bias due to its limited size [28]. These findings underscore a dynamic shift in OSA prevalence among the HF population across the analyzed years and emphasize the need for caution when interpreting results from studies with smaller sample sizes. 

Interestingly, the prevalence of OSA in HF patients appears to vary depending on the type of HF, but these differences are not significant. However, the studies consistently show that HFrEF and HFmrEF patients tend to have higher rates of sleep-disordered breathing, particularly CSA. On the other hand, HFpEF patients exhibit a higher prevalence of OSA [22,24,27,29,40,57,58,59,60]. Additionally, it is noteworthy that the underlying etiology of HF may also influence the prevalence of sleep-disordered breathing, with ischemic and hypertensive groups having higher rates of SDB compared to valvular and arrhythmic groups [21]. Moreover, the studies suggest that OSA patients with HF have higher LVEF values than CSA patients, indicating potential differences in the pathophysiology and mechanisms of these two types of sleep-disordered breathing in HF [20]. In instances of right heart failure, fluid accumulation in the body, including the cervical region, leads to edema. This, in turn, contributes to an escalation in upper airway obstruction.

Furthermore, the inadequacy of the right ventricle impairs the circulation of blood within the pulmonary vessels, culminating in reduced perfusion of the lung tissue. Consequently, there is a decline in oxygenation levels, exacerbating desaturation during episodes of apnea. Conversely, left heart failure prompts the occurrence of pulmonary edema, impacting the optimal perfusion of the alveoli and the ensuing gas exchange process. This, in effect, leads to a reduction in oxygen (O_2_) levels in the blood and an elevation in carbon dioxide (CO_2_) levels. The malfunction of the left ventricle further results in compromised renal perfusion, thereby fostering the development of hypertension and heightened fluid retention within the body, consequently exacerbating generalized edema.

In summary, the findings from these studies underscore the importance of considering sleep-disordered breathing, particularly OSA, in the management and treatment of heart failure patients. The data from the cited studies collectively emphasize the strong association between HFpEF and OSA. The prevalence of OSA in HFpEF patients is significant and highlights the need for routine screening and management of OSA in this population. There is no screening program for OSA in HF patients, and it is primarily attributed to the elevated costs associated with polysomnography and the constrained accessibility of sleep centers, even within well-developed regions. Early diagnosis and appropriate interventions for OSA in HF patients may play a role in improving patient outcomes and quality of life. However, further research is warranted to understand better the mechanisms and implications of sleep-disordered breathing in different types of heart failure and its impact on patient prognosis and management strategies.

### 4.2. The Impact of Positive Airway Pressure Therapy in Heart Failure and Obstructive Sleep Apnea Patients

Sleep-disordered breathing presents a potent stimulant for the upregulation of adrenergic activity. Disrupted sleep patterns and episodes of intermittent hypoxia can potentially initiate excessive sympathetic activity, oxidative stress, vascular inflammation, endothelial dysfunction, arterial stiffness and hypercoagulation [59]. Following the American Academy of Sleep Medicine guidelines, positive airway pressure therapy (CPAP, Auto-PAP and, BiPAP, bilevel positive airway pressure) is recommended in adult patients with OSA [2]. Considering the multifactorial benefits and its targeting of shared pathophysiological pathways in heart failure and obstructive sleep apnea, evaluating the impact of PAP therapy on HF outcomes becomes crucial. CPAP has the strongest evidence for a beneficial cardiovascular effect [61]. Investigations involving PAP treatment have indicated that effective therapy could alleviate the heightened sympathetic activity observed in patients with OSA [62,63]. Studies have demonstrated that each occurrence of breathing cessation during sleep triggers significant elevations in muscle sympathetic nerve activity (MSNA) among individuals with OSA. Comparatively, HF patients with coexisting OSA have demonstrated an increase of 11 bursts per 100 heartbeats in MSNA compared to those without sleep apnea [64]. Notably, a subanalysis of a randomized controlled trial revealed a reduction of 12 bursts per 100 heartbeats in patient MSNA following CPAP therapy, underscoring the potential of distinct sympathoexcitatory mechanisms (HF and OSA) to synergistically heighten MSNA through additive summative effects. 

A moderate confirmation level suggests that OSA is associated with increased serum and plasma inflammatory cytokines, oxidative stress indicators, adhesion molecules, adipose tissue hormones, and abnormal lipid profiles, which can be reduced with PAP treatment [65]. Additionally, PAP may help to maintain sinus rhythm after ablation or electrical cardioversion in patients with atrial fibrillation [66]. Moreover, analyzed studies reported reductions in NT-proBNP concentration after follow-up in HFpEF patients treated with PAP [22,29]. However, it is worth noting that the reduction in NT-proBNP concentration was less significant in patients with OSA compared to non-OSA patients, indicating that OSA may have a modifying effect on the response to treatment. Studies with HFrEF patient groups and PAP treatment also showed a consistent trend toward improvement in LVEF. The LVEF changes suggest that PAP therapy might positively impact cardiac function and improve outcomes in HF patients with coexisting OSA. Gupta et al. also assessed the correlation between the severity of OSA measured by the AHI and diastolic dysfunction in HFpEF patients [22]. The study found a positive correlation between AHI severity and the degree of diastolic dysfunction. This suggests that the presence and severity of OSA may be associated with worsening diastolic function in HFpEF patients.

When analyzing HFrEF patients, studies consistently show significant reductions in the AHI and arousal index after PAP treatment [30,31,32,33,35,37,38]. These improvements indicate successful management of OSA and relief of sleep-disordered breathing in HF patients. Additionally, PAP therapy leads to significant enhancements in desaturation index, LOS, and MOS levels, promoting better sleep quality and increased oxygenation during sleep. Most importantly, studies indicated that PAP treatment was associated with reduced daytime systolic blood pressure and heart rate in HF patients with OSA [30,31,38] among the observational studies and RCTs included in a well-summarized review by Peker et al. PAP treatment significantly reduces blood pressure, especially nocturnal, in OSA patients. Lowering blood pressure is crucial for patients with HF to reduce the workload afterload, prevent cardiac strain, minimize fluid retention, and improve coronary blood flow [67].

Moreover, maintaining a low heart rate reduces myocardial oxygen demand, enhances diastolic filling, optimizes cardiac output, and improves synchronization [68]. A clinical trial conducted on individuals with HFrEF, known as the Ivabradine and Outcomes in Chronic Heart Failure (SHIFT) study, demonstrated the advantageous effects of ivabradine in HF patients with heart rates exceeding 70 beats per minute (bpm) persisted even when patients were already undergoing recommended therapeutic approaches, including beta-blocker therapy [69]. The incidence of major adverse cardiovascular events, such as hospitalization for heart failure and cardiovascular-related mortality, exhibited a noteworthy decrease in the ivabradine-treated group compared to the placebo-treated group. This reduction was particularly prominent among those participants with initially higher baseline heart rates. Additionally, these findings are crucial as hypertension and increased heart rate are common complications in HF, and PAP therapy might play a role in mitigating further cardiovascular risk among OSA patients with heart failure at baseline. 

Age and BMI are significant determinants of LVEF improvement in HF patients with OSA after PAP treatment [30]. Younger patients with obesity demonstrated a higher degree of improvement in LVEF. These observations highlight the importance of patient-specific factors in predicting the response to PAP therapy and suggest that younger, obese patients may benefit more from the intervention. Additionally, several studies indicated that PAP treatment is associated with improved quality of life, NYHA functional class, and daytime sleepiness in HF + OSA patients [31,32,36]. These improvements suggest that PAP may have broader benefits beyond cardiovascular parameters, enhancing these patients’ overall well-being and functional status.

Moreover, in contemporary medical practice, assessing the treatment-related costs and the potential risk of hospitalization in OSA + HF patients has become essential, particularly considering that the HF population tends to have a higher frequency of hospital visits than the general population. Furthermore, it is crucial to investigate whether PAP treatment could contribute to reducing the aforementioned utilization of medical resources in individuals with OSA and HF. Cistulli et al. found that good adherence to PAP therapy resulted in significant improvements in healthcare utilization [39]. Adherent patients experienced reduced hospitalization rates, emergency room visits, and cardiovascular hospitalizations compared to non-adherent patients. Additionally, adherent patients had lower total healthcare costs. Moreover, adherence to PAP therapy in HFrEF patients was associated with reduced healthcare resource utilization, including decreased emergency room visits and healthcare costs [41]. These findings highlight the potential economic benefits of PAP treatment, which may lead to cost-effective management of this patient population.

### 4.3. The Heart Failure Medications on Sleep Parameters: Correlation and Potential Mechanisms

Worsening of HF symptoms can elevate the tendency to obstructive and central apneas. HF can potentially worsen or unmask latent OSA through heightened upper airway instability, particularly during supine sleep due to cervical venous congestion [70]. Research has demonstrated a link between volume redistribution during sleep and AHI in HF patients with OSA [71]. Increased volume load could lead to cervical venous congestion, thus aggravating OSA. Consequently, optimizing HF therapy emerges as the pivotal approach, as it diminishes preload and interstitial lung pressure, thus mitigating the hyperventilation that drives OSA. Preload reduction concurrently alleviates cervical venous congestion and upper airway instability. Given the fluid retention and rostral fluid shift in HF patients, interventions aimed at reducing intravascular volume and venous congestion hold promise in alleviating the severity of both OSA and CSA. 

Pharmacological intervention is a cornerstone in HF management, guided by established protocols. Beta-blockers and ACE inhibitors enhance cardiac output and confer symptomatic relief in OSA [72]. Diuretics effectively curtail OSA severity by impeding fluid retention and curtailing fluid translocation to the oral cavity [73]. For instance, a three-day regimen of spironolactone and furosemide heightened upper airway caliber and decreased AHI (*p* < 0.001) in individuals with diastolic HF and severe OSA [74]. Addressing HF complications warrants particular attention. Pharmacotherapy for HF offers a beneficial impact on OSA by mitigating volume shifts and lung and cervical region volume overload. Cardiac resynchronization therapy has been observed to ameliorate CSA in congestive heart failure patients by reducing AHI. However, significant reductions are yet to be found in subjects with OSA [75]. These initial investigations have occurred against notable transformations in heart failure treatment, leading to improved prognoses in HF.

American Heart Association and European Society of Cardiology new guidelines have added a class of diabetes drugs called SGLT-2 inhibitors (empagliflozin, dapagliflozin) to the list of treatments for heart failure [14,15]. Canagliflozin, ertugliflozin and sotagliflozin are other SGLT2 inhibitors. Conducting studies on the effects of new pharmacotherapy, SGLT2 inhibitors and sacubitril/valsartan in patients with OSA and HF is paramount due to several compelling reasons. These medications represent novel therapeutic approaches in HF treatment, and their potential benefits extend beyond cardiovascular parameters. The SGLT2 inhibitors have gained prominence in the treatment of both HF and type 2 diabetes. Their multifaceted effects encompass cardiovascular benefits, renal protection, and metabolic improvements. Since sleep disturbances are intricately linked to metabolic dysregulation and cardiovascular dysfunction, studying the impact of SGLT2 inhibitors on sleep quality and OSA parameters in HF patients becomes pivotal. The proven positive effects of SGLT2 inhibitors on sleep in diabetes patients underscore the need to explore their potential benefits in the HF population, shedding light on yet unexplored avenues for enhancing patient well-being. When analyzing the effects of SGLT2-i on OSA incidence, Wojeck et al. reported a 48% relative risk reduction in the development of OSA in HF patients treated with ertugliflozin compared to placebo [42]. This finding suggests a potential protective effect of this SGLT2 inhibitor against the development of OSA in HF patients. Additionally, an analysis of the EMPA-REG OUTCOME trial revealed that patients with OSA were at increased risk of cardiovascular and kidney events and higher all-cause mortality [43]. Interestingly, treatment with empagliflozin was associated with a lower likelihood of developing new-onset OSA. These findings suggest a potential benefit of empagliflozin in reducing the risk of OSA and improving outcomes in HF patients with coexisting OSA. Given the constrained availability of data regarding SGLT2 inhibitors in individuals with heart failure and obstructive sleep apnea, our inclusion criteria encompassed studies involving these medications among patients with OSA and other conditions, e.g., diabetes, where more comprehensive data were obtained. Small prospective studies on dapagliflozin indicated potential improvements in sleep parameters in obese type 2 diabetes (T2D) patients with OSA [44,45]. The first study showed a significant improvement in the oxygen desaturation index in the moderate/severe SDB group after dapagliflozin treatment. However, the second study’s small sample size limits the conclusions that can be drawn. Sawada et al.’s retrospective study demonstrated that SGLT2 inhibitors, including dapagliflozin, were associated with weight reduction and improvements in AHI in T2D patients with OSA [46]. However, severe OSA may attenuate the AHI improvement with increasing body weight reduction, suggesting the importance of personalized approaches in this population. Additional evidence of the favorable impact on the pathophysiology of OSA arises from the established influence of SGLT2 inhibitors on visceral and subcutaneous adipose tissue, as demonstrated in previous studies [76,77,78]. This phenomenon is exemplified in animal models of type 2 diabetes mellitus and metabolic syndrome, where the alteration in energy substrates from carbohydrates to lipids results in heightened lipolysis and beta-oxidation of fatty acids [79,80,81]—changes that contribute to the aforementioned positive effects. SGLT2i have also exhibited efficacy in countering liver steatosis in individuals and animals with T2DM [82,83,84,85,86,87]. Notably, the SGLT2 inhibitor, canagliflozin, has been proven to decrease the accumulation of epicardial fat [88], a factor closely linked to coronary heart disease [89,90]. There have been suggestions that SGLT2 inhibitors might yield beneficial outcomes for individuals with obstructive sleep apnea due to a fascinating, albeit debated, mechanism [91]. This mechanism involves the inhibition of leptin activation [92], a hormone found at elevated levels in individuals with OSA [93,94]. Indeed, reinforcing this notion, a recent meta-analysis involving ten randomized controlled trials highlighted that the use of SGLT2i in individuals with type 2 diabetes mellitus was linked to reductions in circulating leptin levels and increases in adiponectin levels [95]. There is a potential link between leptin and obstructive sleep apnea, although the relationship is complex and not fully understood. Leptin concentration tends to be higher in individuals with more adipose tissue, and obesity is associated with leptin resistance. The resistance may contribute to disruptions in appetite regulation and potentially impact the regulation of breathing during sleep. Moreover, hypoxia can influence the production and release of various hormones, including leptin. Increased sympathetic activity in OSA individuals can impact hormonal regulation, including leptin production and signaling. Disrupted sympathetic activity due to OSA might influence leptin’s actions and further complicate metabolic pathways [96]. The results of the ongoing DAHOS trial will provide further insights into the potential role of dapagliflozin in managing OSA and heart failure in this specific patient population. 

Another new medicament in HF pharmacotherapy, sacubitril/valsartan, was assessed in several studies with OSA patients. Sacubitril/valsartan, a neprilysin inhibitor combined with an angiotensin receptor blocker, has demonstrated remarkable efficacy in HF therapy. S/V acts by inhibiting neprilysin, thereby preventing the degradation of natriuretic peptides. This, in turn, amplifies their natriuretic and vasodilatory impacts, leading to decreased pulmonary congestion [97,98]. The treatment also positively affects cardiac reverse remodeling, a phenomenon linked to improved LVEF, potentially augmenting cardiac output [99,100]. These combined effects can enhance respiratory efficiency and optimize gas exchange.

Furthermore, the treatment may influence the chemoreflex by diminishing pulmonary stretch receptor activation and enhancing peripheral chemoreceptor perfusion [101]. Another conceivable outcome of increased cardiac output is reduced circulation time, which limits the chemoreflex system’s capacity to recognize and react to fluctuations in CO_2_ levels [102]. Lastly, this medication has demonstrated the capacity to mitigate the upward shift of fluids towards the head that typically occurs when an individual is in a reclined position [103]. To the best of our knowledge, Fox et al. identified the first case of a 71-year-old male with heart failure and sleep-disordered breathing, in which administering sacubitril/valsartan therapy was linked to enhanced cardiac function, evidenced by a reduction in NT-proBNP levels and an improvement in LVEF and a substantial decrease in the AHI. This instance marks the inaugural presentation of amelioration in both HF and SDB subsequent to the initiation of SV treatment [104]. In another analyzed study, we found minimal changes in the AHI and total sleep time after eight weeks of S/V treatment, suggesting that these medications might not have a significant impact on OSA parameters [48]. However, the effects of a 6-month therapy with sacubitril/valsartan on hemodynamic, sleep and metabolic parameters demonstrated significant improvements in AHI, oxygen desaturation index, and time spent with oxygen saturation below 90% [49].

Additionally, there were significant decreases in BMI, NT-proBNP concentration, and improvements in LVEF. Moreover, patients primarily exhibiting a central pattern of irregular breathing and an obstructive pattern of breathing showed a decrease in AHI. These findings from the ENTRESTO-SAS trial suggest that sacubitril/valsartan might have a positive impact on sleep parameters in patients with both CSA and OSA [50]. Additionally, the medication was associated with improved cardiac biomarkers and left ventricular function, further supporting its potential benefits in managing sleep apnea and heart failure with reduced ejection fraction. However, further studies with larger sample sizes and longer follow-up periods are necessary to confirm these observations and provide more definitive evidence. 

Incorporating an investigation into the effects of SGLT-2i and sacubitril/valsartan on sleep parameters aligns with contemporary patient-centered care, where a holistic approach encompasses the management of cardiac function and the overall well-being of HF patients. Recognizing any modifications in sleep patterns due to these medications can aid healthcare providers in optimizing treatment plans and improving patient outcomes. 

### 4.4. OSA and HF—Clinical Relevance, Clinical Practice and Patient Care

The relationship between OSA, HF, and cardiovascular medications (like SGLT2i and sacubitril/valsartan) significantly impacts clinical practice and patient care. Incorporating this correlation into healthcare strategies may enhance the efficiency of screening and treating patients with the above-mentioned interconnected conditions. Regarding screening strategies, it is important to identify OSA in HF patients, particularly those with preserved ejection fraction, due to the high prevalence of OSA in this population. Routine OSA screening and using tools such as the STOP-BANG questionnaire or portable sleep study devices may facilitate early detection and successful intervention. Additionally, acknowledgement of common risk factors (obesity, hypertension, daytime sleepiness) shared between OSA and HF may guide clinicians to perform OSA evaluation for particular patients. Managing HF should include not only traditional HF medications but also treatments addressing comorbid conditions like OSA. Effective management of OSA may contribute to the improvement of HF outcomes.

Furthermore, cardiovascular medications like SGLT2i and sacubitril/valsartan have displayed promise in enhancing cardiac function among HF patients. Thus, clinicians should consider incorporating these medications into their HF + OSA management strategy. Exploring combination therapy is essential, as these medications may offer synergistic benefits for HF patients with OSA. Apart from the primary indications, the above-mentioned pharmacotherapy may also be used to reduce OSA-related cardiovascular risks. In patient-centered care, recognizing the diversity among HF patients is crucial. Personalized treatment plans should include the presence of OSA and the choice of cardiological medications based on individual patient’s characteristics and comorbidities. The role of effective follow-up is worth emphasizing. Patients with HF and OSA may benefit from long-term monitoring of both conditions to assess treatment effectiveness. Regular follow-up appointments may help clinicians adjust treatment strategies as needed to optimize patients’ outcomes. Lastly, education of the patients is a key to therapeutic success. Patients should know the interplay between OSA, HF, and cardiological medications. Overall outcomes of the therapy may be significantly improved by encouraging patients to report sleep-related symptoms and engage in discussions with their healthcare providers about treatment options. 

In conclusion, this systematic review is a valuable effort to advance our understanding of the intricate relationship between OSA and HF. By exploring the bidirectional influences between these conditions and examining the impact of innovative pharmacotherapies of HF on sleep parameters, the authors aspire to contribute significantly to the expanding knowledge base in this field. This undertaking is pivotal in guiding evidence-based clinical decisions, fostering multidisciplinary approaches, and ultimately improving the quality of life for individuals grappling with the complexities of both OSA and HF.

Importantly, the current AASM recommendations for optimal sleep breathing disorders treatment include, among others, body mass reduction, PAP therapy, oral appliances, and surgical methods. Medicaments used in the treatment of heart failure do not reverse airway obstruction. Analyzing data from randomized controlled trials might only enable selecting an appropriate method of pharmacotherapy and sleep apnea management dedicated to patients co-suffering from HF and OSA. Optimized therapy could potentially and maximally reduce OSA and HF complications, extend life expectancy, improve the quality of life and sleep, and reduce the risk of hospitalization. Nevertheless, treating patients with multi-chronic conditions should target all the diseases’ causes. Patients with comorbid OSA and HF should obtain proper HF treatment and OSA management. 

Despite the promising results, the current systematic review has some limitations that should be acknowledged. Firstly, substantial heterogeneity across included studies, stemming from differences in study populations and outcome measures, may hinder the comprehensive pooling of results for meaningful analysis. Additionally, randomized controlled trials are limited in the context of novel HF pharmacotherapy on OSA outcomes. Including different study types allowed for a more comprehensive exploration of this intricate relationship between pharmacotherapy and sleep parameters in HF patients with coexisting OSA. Secondly, the review’s potential language bias, resulting from the restriction of the search to the English language, raises the possibility of omitting relevant studies conducted in other languages, introducing a source of bias into the analysis. Thirdly, the inherent publication bias in the literature, wherein studies with significant findings are more likely to be published, might lead to an unintentional overrepresentation of positive results in the review. Lastly, the limited availability of long-term data in most of the included studies might impede a thorough understanding of the potential long-term effects of interventions, particularly if the majority of studies are short-term in nature. These limitations should be considered when interpreting the findings and implications of the review. Therefore, additional large-scale, well-controlled clinical trials are necessary to confirm and further investigate the effects of these medications on sleep parameters and clinical outcomes in HF patients.

Overall, the evidence presented in these studies underscores the importance of recognizing and managing OSA in patients with heart failure to optimize their overall outcomes and quality of life. Multidisciplinary approaches that incorporate cardiovascular and sleep medicine specialists may benefit the comprehensive management of these patients. Future research will likely provide more insights and pave the way for more effective therapeutic strategies for heart failure patients with coexisting obstructive sleep apnea.

## 5. Conclusions

The evidence presented in the above-mentioned studies strongly supports the association between heart failure and obstructive sleep apnea, highlighting the need for early detection and appropriate management of sleep-disordered breathing in heart failure patients. The data suggest that implementing effective interventions for obstructive sleep apnea, such as PAP treatment, might lead to significant improvements in sleep parameters, cardiac function, and overall patient well-being. Furthermore, using PAP in heart failure patients with coexisting OSA can optimize patient outcomes, reduce HF-related hospitalizations, and lower healthcare costs. Additionally, the integration of novel pharmacotherapeutic agents such as SGLT2 inhibitors and sacubitril/valsartan in the treatment regimen for heart failure holds promise for ameliorating sleep parameters in patients with OSA + HF. Exploring these innovative therapeutic modalities offers the potential to reveal favorable effects on sleep disruptions associated with OSA in the context of heart failure pathology. Multidisciplinary collaboration between cardiovascular and sleep medicine specialists is most likely beneficial in providing comprehensive care to heart failure patients with coexisting obstructive sleep apnea.

## Figures and Tables

**Table 1:** The PICO’s question.

What Is the Prevalence of OSA in HF Patients?/How Do Sleep and Cardiac Parameters Change after PAP Therapy in These Patients?/Does New Cardiological Pharmacotherapy (SGLT2i and Sacubitril/Valsartan) Play a Role in the Treatment of OSA?	
The population	Patients with OSA and HF (OSA + HF)/patients with OSA and SGLT2i or sacubitril/valsartan in treatment
The indicator	AHI, LVEF, NT-proBNP concentration.
The control	Groups of patients without OSA or patients with other SDB/ patients without PAP treatment/patients without SGLT2i or sacubitril/valsartan in the treatment
The outcome	The difference in AHI/LVEF/NT-proBNP/BNP concentration
The study design	Peer-reviewed English articles. Adult (>18 years) human subjects. Case–control studies, randomized control trials, and observational studies.

Abbreviations: OSA, obstructive sleep apnea; SDB, sleep-disordered breathing; HF, heart failure; PAP, positive airway pressure; SGLT2i, sodium/glucose cotransporter-2 inhibitors; AHI, apnea-hypopnea index; LVEF, left ventricle ejection fraction; NT-proBNP, N-Terminal pro-Brain Natriuretic Peptide.

**Table 2:** The characteristics and results of chosen studies populations.

Author, Year	N	Sex, M/F	Age, Years	BMI, kg/m^2^	EF, %	AHI	OSA Prevalence, %	Overall OSA Prevalence, %	SDB Prevalence, %
Kalaydzhiev et al., 2023 [28]	100 screened; 61 SDB; 50 OSA; 11 CSA	32/29	66.2 ± 9.1 OSA; 66.1 ± 11.9 CSA	38.5 ± 7.1 OSA; 31.9 ± 4.5 CSA	49.6 ± 8.5 OSA; 41.8 ± 11.4 CSA	41.8 ± 23.2 OSA; 37.7 ± 12.6 CSA	81.97 (50/61)	50 (50/100)	61 (61/100)
Wang et al., 2022 [20]	252, 36 r; 43 mr; 173 p	134/118	70.1, 68.3 ± 12.6 r; 65.0 ± 14.7 mr; 71.8 ± 11.8 p	24.8 24.0 (21.1, 27.3)r; 24.8 (23.1, 27.0)mr; 24.5(22.0, 26.9)p	<40 r; 40–50 mr; ≥50 p	26.4 ± 16.2 r; 26.1 ± 15.2 mr; 14.4 ± 15.6 p *◊	42 (15/36)r; 47 (20/43)mr; 49 (85/173)p	48 (120/252)	86 (31/252) r; 86 (37/252) mr; 62 (108/252) p *◊
Wang et al., 2022 [21]	248, 89 I; 43 H; 36 M; 27 V; 53 A	132/116	70.4 ± 12.4, I: 73.0 (66.0–81.5); H: 75.0 (68.0–82.0); M: 67.0 (56.0–75.0); V: 73.0 (62.0–82.0); A: 70.0 (61.5–79.0)	24.5, I: 24.0 (21.9–26.7); H: 25.5 (22.9–28.7); M: 24.3 (21.2–27.2); V: 23.9 (20.1–25.8); A: 24.8 (22.2–27.8)	ND	I: 18.3 (5.0–31.4); H: 12.8 (6.1–28.0); M: 20.3 (9.3–34.5); V: 6.6 (1.7–22.5) ◊; A: 6.9 (3.6–20.5)	ND	47.6, 38 (42.7%)I; 31 (72.1%)H; 13 (36.1%)M; 10 (37.0%)V; 26 (49.1%)A	70.6 (175/248)
Gupta et al., 2020 [22]	50 (25P/25C)	40/10	58.4 + 9.8 OSA; 60 + 10 CSA	27.9 + 1.6 OSA; 29.4 + 0.6 CSA	55.84 + 2.01 ◊ SDB; 52.08 + 3.24 ◊ noSDB	9.9 + 4.2 ◊ SDB; 3.7 + 1.1 ◊ noSDB	52 (13/25)	26 (13/50)	32 (16/50)
Arzt et al., 2017 [23]	9221screened, 1557SDB; 452 OSA; 624 OSA + CSA; 481 CSA	1353/204	66 ± 11 OSA; 69 ± 10 OSA + CSA; 69 ± 10 CSA	31 ± 6 OSA; 29 ± 5 OSA + CSA; 28 ± 4 CSA	35 ± 8 OSA; 34 ± 8 OSA + CSA; 32 ± 8 CSA	37 ± 19 OSA; 36 ± 16 OSA + CSA; 38 ± 15 CSA	29 (452/1557)	4.90 (452/9221)	16.89 (1557/9221)
Herrscher et al., 2011 [27]	115, 62 OSA, 31 CSA, 22 noSDB	91/24	62.0 ± 9.7; 62.46 ± 9.2 OSA; 62.26 ± 10.6 CSA; 60.26 ± 10.0 noSDB	30.2 ± 6.0 OSA; 28.4 ± 4.2 CSA; 27.1 ± 4.8 noSDB	40.4 ± 13.2 OSA; 34.0 ± 12.5 CSA; 37.3 ± 12.0 noSDB	25.06 ± 21.7 OSA #◊; 26.86 ± 13.1 CSA #◊; 2.36 ± 1.5 noSDB	66.67 (62/93)	53.91 (62/115)	80.87 (93/115)
Bitter et al., 2009 [24]	244, 72 CSA;97 OSA;75 noSDB	157/87	65.3 ± 1.4; 66.9 ± 2.4 CSA ‡; 66.8 ± 1.9 OSA †; 61.6 ± 3.3 noSDB	29.3 ± 0.9 CSA ‡; 29.3 ± 1.1 OSA†; 26.42 ± 1 noSDB	>55	impaired relaxation: 15.0 ± 3.6; pseudonormal: 20.0 ± 3.3 †; restrictive: 23.4 ± 6.2 ‡	57.4 (97/169)	39.75 (97/244)	69.3 (169/244)
Yumino et al., 2009 [26]	218, 56 OSA; 45 CSA;117 M-NSA	168/50	55.66 ± 12.7 56.36 ± 12.1 OSA; 60.46 ± 8.9 CSA; 53.46 ± 13.6 M-NSA	29.26 ± 5.3 31.0 ± 5.0 ^ OSA; 27.8 ± 5.4 CSA **; 28.9 ± 5.3 M-NSA	25.76 ± 9.1 OSA; 21.36 ± 9.5 CSA; 25.56 ± 10.3 M-NSA	33.6 ± 14.5 ^ OSA; 34.8 ± 15.6 ‘ CSA; 6.8 ± 3.9 M-NSA	55.45 (56/101)	25.69 (56/218)	46.33 (101/218)
Oldenburg et al., 2007 [16]	700, 253 OSA; 278 CSA; 169 noSDB	139/561	65.02 ± 9.5 OSA ^a^; 65.86 ± 10.5 CSA^a^; 61.45 ± 11.0 noSDB	27.84 ± 4.7 OSA^a^; 26.30 ± 4.1 CSA ^b^; 25.77 ± 3.7 noSDB	29.3 ± 2.6 OSA; 27.4 ± 6.6 CSA ^a^; 28.2 ± 7.3 noSDB	18.45 ± 13.3 ^a^ OSA; 30.15 ± 15.2 ^a,b^ CSA; 2.28 ± 1.6 noSDB	48.21 (256/531)	36 (256/700)	76 (531/700)
Chan et al., 1997 [25]	20, 11 SDB, 9 noSDB	7/13	65 ± 6.0 7.3 ± 1.3 SDB; 7.2 ± 0.8 noSDB	ND	28 ± 3.2 29.1 ± 4.2 SDB; 27.6 ± 1.3 noSDB	19.5 ± 10.8 SDB ◊; 3.9 ± 3.5 noSDB ◊	63.64 (7/11)	35 (7/20)	55 (11/20)

Abbreviations: r, reduced ejection fraction; mr, mildly-reduced ejection fraction; p, preserved ejection fraction; ND, no data; ◊, significant difference, *p* < 0.05; *, *p* < 0.05, p/mr group vs. r group and *p* < 0.05, p group vs. mr group; M/F, n Male/Female; I, ischemic group; H, hypertensive group; M, myocardial group; V, valvular group; A, arrhythmic group; EF, ejection fraction; M-NSA, mild or no sleep apnea; OSA, obstructive sleep apnea; CSA, central sleep apnea; P, patients with sleep disordered breathing; C, controls; SDB, sleep disordered breathing; noSDB, no sleep-disordered breathing; #◊, *p* < 05, OSA/CSA vs. noSDB; †, *p* < 0.05 OSA vs. noSDB; ‡, *p* < 0.05 CSA vs. noSDB; ^, *p* < 0.05 M-NSA vs. OSA; ‘, *p* < 0.05 M-NSA vs. CSA; **, *p* < 0.05 OSA vs. CSA; ^a^, *p* < 0.05 vs. no SDB; ^b^, *p* < 0.05 vs. OSA; overall OSA prevalence, number (n) of OSA/n all study participants; OSA prevalence, n OSA/n SDB participants. Data is presented as the percentage of cohort or mean ± standard deviation or median.

**Table 3:** The characteristics and results of a chosen study population with OSA and HFpEF.

Author, Year	N	Sex, M/F	Age, Years	EF, % Pre	EF, % Post	OSA Prevalence, %	CPAP Adherence	AHI Pre	AHI Post	BNP Pre, pg/mL	BNP Post, pg/mL
Arikawa et al., 2016 [29]	58	31/19	66 ± 15 (OSA) 65 ± 11 (nOSA)	61 ± 5 (OSA) 63 ± 9 (nOSA)	ND	67% (39/58)	ND	ND	ND	444 (233–752) (OSA) 316 (218–703) (nOSA)	1 m: 302 (202–350) (OSA) 212 (180–405) (nOSA) 6 m: 222 (137–324) ◊ (OSA) 76 (38–96) ◊ (nOSA) 12 m: 123 (98–197) ◊ (OSA) 52 (38–76) ◊ (nOSA) 36 m: 115 (64–174) ◊ (OSA) 56 (25–74) ◊ (nOSA)

Abbreviations: ND, no data; ◊, significant difference; M/F, Male/Female; EF, ejection fraction; OSA, obstructive sleep apnea; nOSA, no obstructive sleep apnea. Data is presented as a percentage of cohort or mean ± standard deviation or median.

**Table 4:** The characteristics and results of chosen OSA and HFrEF studies.

Author, Year	N	Sex, M/F	Age, Years	Ef, % Pre	EF, % Post	CPAP Duration	AHI pre	AHI Post	NT-proBNP Pre	NT-proBNP Post
Naito et al., 2022 [30]	55 OSA	52/3	60.7 ± 12.2	37.2 ± 9.8 ◊	43.2 ± 11.7 ◊	1 m	45.3 ± 16.1 ◊	5.4 ± 4.1 ◊	ND	ND
Kaneko et al., 2003 [31]	24 12 c/12 p	21/3	55.2 ± 3.6 c 55.9 ± 2.5 p	28.5 ± 1.8 c 25.0 ± 2.8 p	ND	1 m	45.2 ± 5.3 c 37.1 ± 6.4 p◊	44.7 ± 6.8 c 8.3 ± 2.8 p◊	ND	ND
Mansfield et al., 2004 [32]	55 27 c/28 p	52/3	57.5 ± 1.6 c 57.2 ± 1.7 p	33.6 ± 2.6 c 37.6 ± 2.5 p Δ1.5 ± 1.4 ◊	35.1 ± 3.1 c 42.6 ± 0.3 p Δ5.0 ± 1.0 ◊	3 m	26.6 ± 4.5 c 25.0 ± 4.1 p Δ−8.4 ± 3.6 ◊	18.2 ± 2.8 c 2.9 ± 0.8 p Δ−21.1 ± 3.8 ◊	ND	ND
Fox et al., 2023 [33]	58 33 c/25 pa	51/7	64.9 ± 10.1 c 67.4 ± 9.8 pa	36.31 ± 6.91 c 39.29 ± 6.51 p◊	39.23 ± 9.41 c 44.35 ± 8.96 p◊	6 m	35 ± 13 c 34 ± 17 p◊	33 ± 20 c 9 ± 8 p◊	ND	ND
Kim et al., 2019 [34]	52 26 c/26 p	48/4	48.8 ± 10.7 c 49.1 ± 11.4 p	64 ± 6 c 66 ± 5 p	64 ± 6 c 65 ± 6 p	3 m	53.4 ± 20.5 c 64.2 ± 20.5 p	ND	ND	ND
Gilman et al., 2008 [35]	19 7 c/12 p	17/2	58.1 ± 7.1 c 56.7 ± 8.0 p	30.4 ± 10.5 c 26.4 ± 10.3 p◊	29.5 ± 6.3 c 34.8 ± 8.3 p◊	1 m	41 ± 13 c 30 ± 15 p◊	37 ± 18 c 7 ± 6 p◊	ND	ND
Servantes et al., 2018 [36]	65 18 c/17 e/15 p/ 15 e,p	43/65	57 ± 8 c 51 ± 9 e 57 ± 7 p 53 ± 10 e,p	29 ± 6 c 31 ± 5 e 31 ± 6 p 33 ± 5 e,p	ND	3 m	29 ± 17 c 28 ± 17 e◊ 32 ± 25 p◊ 25 ± 15 e,p◊	31 ± 14 c 18 ± 12 e◊ 8 ± 11 p◊ 10 ± 16 e,p◊	ND	ND
Egea et al., 2008 [37]	60 32 c/28 p	56/60	63 ± 1.6 c 64 ± 0.9 p	28.1 ± 1.5 c 28.0 ± 0.5 p◊	28.1 ± 1.7 c 30.5 ± 0.8 p◊	3 m	35.3 ± 3.1 c 43.7 ± 4.4 p◊	28.0 ± 4.6 c 10.8 ± 2.2 p◊	ND	ND
Ryan et al., 2005 [38]	18 8 c/10 p	16/2	60.3 ± 4.1 c 57.6 ± 2.2 p	34.1 ± 3.0 c 27.6 ± 3.4 p◊	29.6 ± 3.1 c 34.3 ± 2.8 p◊	1 m	57.9 ± 5.50 c 29.3 ± 4.8 p◊	56.2 ± 5.3 c 6.1 ± 1.1 p◊	ND	ND

Abbreviations: OSA, obstructive sleep apnea; ND, no data; ◊, significant difference; M/F, Male/Female; EF, ejection fraction; p, participants receiving CPAP; pa, participants receiving APAP; c, controls; m, month(s); e, exercise; e,p, exercise, and CPAP. Data is presented as a percentage of cohort or mean ± standard deviation or median.

**Table 5:** Effect of PAP adherence on costs, hospitalizations, and ER visits in OSA and HFpEF/HFrEF.

Author, Year	N	Sex, M/F	Age, Years	PAP Adherence	PAP Usage	Effect of PAP Adherence on Hospitalizations and ER Visits
Cistulli et al., 2023 [39]	4237	1950/2287	64.1	64.1% Adherent (*n* = 1701) Intermediate (*n* = 1250) Nonadherent (n = 1286)	Hours per day: A: 6.8 ± 1.5 ◊ I: 2.9 ± 1.4 ◊ N: 0.4 ± 0.6 ◊ Days per week: A: 6.6 ± 0.5 ◊ I: 3.8 ± 1.7 ◊ N: 0.9 ± 1.2 ◊ Hours per use day: A: 7.2 ± 1.4 ◊ I: 5.4 ± 1.3 ◊ N: 2.9 ± 1.7 ◊	Composite: A: 1.22 ± 2.06; I: 1.88 ± 3.12; N: 1.99 ± 3.21 A-N ◊ A-I ◊ I-N ER: A: 0.89 ± 1.66; I: 1.37 ± 2.54, N: 1.41 ± 2.68 A-N ◊ A-I ◊ I-N All-cause hospitalization: A: 0.33 ± 0.84; I: 0.51 ± 1.23, N: 0.59 ± 1.17 A-N ◊ A-I ◊ I-N ◊ Cardiovascular hospitalization: A: 0.06 ± 0.27, I: 0.13 ± 0.61, N: 0.13 ± 0.47 A-N ◊ A-I ◊ I-N
Malhotra et al., 2023 [41]	3182	2223/959	59.7 ± 11.2	Adherent 39%, (n = 1252); Intermediate 29%, (n = 935); Nonadherent 31%, (n = 995)	Hours per day: A: 6.6 ± 1.5 ◊ I: 2.8 ± 1.4 ◊ N: 0.4 ± 0.6 ◊ Days per week: A: 6.6 ± 0.5 ◊ I: 3.8 ± 1.7 ◊ N: 0.9 ± 1.1◊ Hours per use day: A: 7.1 ± 1.4 ◊ I: 5.4 ± 1.3 ◊ N: 2.9 ± 1.6 ◊	Composite: A: 1.00 ± 1.73; I: 1.30 ± 2.09; N: 1.37 ± 2.56 A-N ◊ A-I ◊ I-N ER: A: 0.71 ± 1.38; I: 0.91 ± 1.65; N: 1.00 ± 2.06 A-N ◊ A-I ◊ I-N All-cause hospitalization: A: 0.29 ± 0.77; I: 0.38 ± 0.93; N: 0.37 ± 0.99 A-N A-I ◊ I-N Cardiovascular hospitalization: A: 0.10 ± 0.43; I: 0.12 ± 0.47; N: 0.12 ± 0.47 A-N A-I I-N
Abdullah et al., 2018 [40]	12,608,637, OSA 653,762; nOSA 11,954,875	5,442,091/7,166,546	OSA 62.5 ± 13.7 nOSA 58.6 ± 20.8	ND	ND	ND

Abbreviations: OSA, obstructive sleep apnea patients; nOSA, no obstructive sleep apnea patients; ◊, statistically significant; M/F, Male/Female; ER, emergency room; A, adherent; I, intermediate; N, non-adherent; A-N, adherent-to-nonadherent; A-I, adherent-to-intermediate; I-N, intermediate-to-nonadherent; ND, no data. Data is presented as a percentage of cohort or mean ± standard deviation.

**Table 6:** Characteristics from SGLT2i studies.

Author, Year	N	Sex, M/F	Age, Years	Rate/1000 Patient- Years	3P-MACE	CV Death	HHF	All-Case Mortality	Incident or Worsening Nephropaty	Changes in Sleep Parame- Ters
Wojeck et al., 2023 [42]	5126 E, 2557 P	69.2(%)	64.3	1.4 E 2.6 P ◊	ND	ND	ND	ND	ND	ND
Neeland et al., 2020 [43]	7020 w/OSA: 4421 Em, 2208 P; 391 OSA: 266 Em, 125 P	5016/2004	63.1 ± 8.6 w/OSA, Em; 63.2 ± 8.9 w/OSA, P; 63.7 ± 7.7 OSA, Em; 63.7 ± 7.3 OSA, P	2.2 E; 4.6 P ◊	490/4687 Em; 282/2333 P ◊	172/4687 Em; 137/2333 P ◊	126/4687 Em; 95/2333P ◊	269/4687 Em; 194/2333 P ◊	525/4687 Em; 388/2333 P ◊; 459/4687 Em; 330/2333 P ◊	ND
Furukawa et al., 2018 [44]	30, 24 mSDB; 6 m-sSDB	20/10	59.0 ± 10.7 mSDB; 58.3 ± 11.7 m-sSDB	ND	ND	ND	ND	ND	ND	3% ODI, baseline: 25.0 ± 3.8; follow-up: 18.5 ± 6.1 ◊
Tang et al., 2019 [45]	36, 18 dapa; 18 w/dapa	22/14	56.10 ± 7.2 dapa; 57.8 ± 10.07 w/dapa	ND	ND	ND	ND	ND	ND	AHI dapa: baseline 37.45 ± 6.04 vs. follow-up 26.72 ± 4.69 ◊; w/dapa: baseline38.11 ± 6.27 vs. follow-up 36.1 ± 4.50; LSpO2: dapa: baseline 84.06 ± 14.58 vs. follow-up 87.16 ± 13.56 ◊; w/dapa: baseline 83.72 ± 13.77 follow-up 84.12 ± 13.83
Sawada et al., 2018 [46]	18	14/4	64 ± 13	ND	ND	ND	ND	ND	ND	AHI baseline: 31.9 ± 18.0; follow-up 18.8 ± 11.5 ◊

Abbreviations: E, patients with ertugliflozin; Em, empagliflozin; dapa, patients with dapagliflozin; w/dapa, patients without dapagliflozin; P, placebo; OSA, obstructive sleep apnea patients; w/OSA, patients without obstructive sleep apnea; ◊, significant difference; M/F, Male/Female; 3P-MACE, 3-point major adverse CV events; HHF, hospitalization for heart failure; mSDB, mild sleep disordered breathing; m-sSDB, moderate-to-severe sleep disordered breathing; ODI, oxygen desaturation index; LSpO2, lowest oxygen saturation, AHI, apnea/hypopnea index; ND, no data. Data is presented as a percentage of cohort or mean ± standard deviation.

**Table 7:** Characteristics from Sacubitril/Valsartan studies.

Author, Year	N	Sex, M/F	Age, Years	Rate/1000 Patient- Years	AHI Pre	AHI Post	N-TproBNP	MOS	ODI
Owens et al., 2021 [48]	140, 70 S/; 70 E	108/32	62.3 ± 8.8 S/V; 64.2 ± 11.6 E	ND	16.3 ± 14.2 S/V; 16.8 ± 14.3 E	15.2 ± 15.6 S/V; 17.6 ± 16.3 E	ND	ND	ND
Pelaia et al., 2022 [49]	132	107/25	67.0 ± 9.8	ND	26.5 ± 10.4	21.7 ± 8.3 ◊	baseline: 1840 (886.0–3378); follow-up: 970.0 (571.3–2870) ◊	baseline: 91.3 ± 1.9; follow-up: 92.0 ± 2.0 ◊	baseline: 18.0 ± 3.7; follow-up: 13.5 ± 4.9 ◊
Jaffuel et al., 2021 [50]	118, 49G1; 27G2; 42G3	96/22	66.00 (56.00–73.00)	ND	24.20 (16.40–43.50) G1 + G2; 22.90 (16.00–43.50) G1; 30.10 (26.40–47.60) G2	20.40 (12.70–31.10) G1 + G2 ◊; 19.20 (12.70–31.10) G1◊; 22.75 (14.60–36.90) G2	G1 baseline: 1811.00 (987.00; 3958.00), follow-up: 1104.00 (391.00; 3075.00) ◊; G2 baseline: 2043.00 (845.0; 3445.00), follow-up: 1351.00 (44.00; 2164.00) ◊; G3 baseline: 852.00 (244.0; 2102.0), follow-up: 591.50 (205.0; 1128.5) ◊	G1 + G2 baseline: 92.30 (91.35–94.55), follow up: 93.05 (91.60–94.70); G1 baseline: 93.00 (91.80–94.60), follow-up: 93.40 (92.20–94.90) G2 baseline: 91.3 (90.00–93.00), follow-up: 91.80 (91.00–92.10)	G1 + G2 baseline: −6.32 ( ± 15.79), follow-up:−6.20 (−12.70 to 0.90) ◊; G1 baseline: 11.90 (7.10–14.65), follow-up: 7.65 (4.90–13.65); G2 baseline: 31.00 (15.30–55.90), follow-up: 24.00 (11.00–45.90)
Wang et al., 2023 [51]	18, 9 OSA, 7 CSA, 2 NB	15/3	66.7 ± 10.7	ND	overall population 20 ± 23 *◊; OSA 14 ± 6 *◊; CSA 36 ± 32 ◊*	overall population 7 ± 7 ◊*; OSA 7 ± 7 ◊*; CSA 7 ± 8 ◊*	baseline 1792.1 ± 1271.3; Three months follow-up 876.9 ± 984.2 ◊	ND	ND
Passino et al., 2021 [52]	51, 15 OSA, 33 CSA	39/12	65 ± 9	ND	overall population: daytime 7 (2–20); nighttime 19 (7–37); 24 h 13 (5–26); OSA: daytime 6 (2–12); nighttime 18 (10–30); 24 h 13 (5–16); CSA: daytime 10 (2–22); nighttime 23 (9–41); 24 h 14 (6–31)	overall population: daytime 3 (0–7) ◊; nighttime 16 (7–23) ◊; 24 h 8 (3–14) ◊; OSA: daytime 1 (0–3) ◊; nighttime 15 (9–27); 24 h 7 (3–8) ◊; CSA: daytime 3 (1–10) ◊; nighttime 16 (7–23) ◊; 24 h 7 (3–16) ◊	baseline 1439 (701–3015); Six months follow-up 604 (320–1268)	ND	ND

Abbreviations: E, patients with enalapril; S/V, patients with sacubitril/valsartan; Em, empagliflozin; dapa, patients with dapagliflozin; w/dapa, patients without dapagliflozin; P, placebo; OSA, obstructive sleep apnea patients; w/OSA, patients without obstructive sleep apnea; CSA, central sleep apnea; NB, normal breathing; ◊, statistically significant; M/F, Male/Female; 3P-MACE, 3-point major adverse CV events; HHF, hospitalization for heart failure; mSDB, mild sleep disordered breathing; m-sSDB, moderate-to-severe sleep disordered breathing; ODI, oxygen desaturation index; MOS; mean oxygen saturation; LSpO2, lowest oxygen saturation, AHI, apnea/hypopnea index; ND, no data; G1, group 1, AHI central ≥ 5/h and AHI obstructive < 15/h; G2: group 2, AHI obstructive ≥ 15/h regardless of the AHI central; G3: group 3, AHI central < 5/h and AHI obstructive < 15/h; *, REI, respiratory events index (events/hour); 24 h, 24-h AHI. Data is presented as a percentage of cohort or mean ± standard deviation or median.

## Supplementary Materials

The following supporting information can be downloaded at: https://www.mdpi.com/article/10.3390/jcm12196139/s1, Table S1: Quality assessment of included studies. Table S2. Effective Public Healthcare Panacea Project—Quality Assessment Tool for Quantitative Studies Dictionary.
