# Supplementary material for: Association between Obstructive Sleep Apnea and Heart Failure in Adults—A Systematic Review
Source: J Clin Med. 2023 Sep 22;12(19):6139. doi: 10.3390/jcm12196139 (PMC10573908; doi:10.3390/jcm12196139)
Supplement: Supplementary file 1 [file jcm-12-06139-s001.zip › jcm-2600181-supplementary.pdf]

Table S1. Quality assessment of included studies.

Author, year	A<br>Selection Bias	B<br>Study Design	C<br>Confounders	D<br>Blinding	E<br>Data Collection Method	F<br>Withdrawals and Dropouts	Global Rating
**Wang et al., 2022** [20]	M	M	S	N/A	S	N/A	Strong
**Wang et al., 2022** [21]	M	M	S	N/A	S	N/A	Strong
**Gupta et al., 2020** [22]	M	M	W	N/A	S	N/A	Moderate
**Arzt et al., 2017** [23]	M	S	M	N/A	S	N/A	Strong
**Oldenburg et al., 2007** [16]	M	M	M	N/A	S	N/A	Moderate
**Bitter et al., 2009** [24]	M	M	M	N/A	M	N/A	Moderate
**Chan et al., 1997** [25]	M	M	M	N/A	M	N/A	Moderate
**Yumino et al., 2009** [26]	S	M	S	N/A	S	N/A	Strong
**Herrscher et al., 2011** [27]	M	M	S	N/A	M	N/A	Moderate
**Kalaydzhiev et al., 2023** [28]	S	M	M	N/A	S	N/A	Strong
**Arikawa et al., 2016** [29]	M	M	S	M	M	W	Moderate
**Naito et al., 2022** [30]	M	M	M	W	M	M	Moderate
**Kaneko et al., 2003** [31]	M	S	S	S	S	M	Strong
**Mansfield et al., 2004** [32]	S	S	S	S	S	S	Strong
**Fox et al., 2021** [33]	S	S	S	S	M	S	Strong
**Kim et al., 2019** [34]	S	S	M	S	M	S	Strong
**Gilman et al., 2008** [35]	S	S	S	S	S	S	Strong
**Servantes et al., 2018** [36]	S	S	M	S	S	S	Strong
**Egea et al., 2007** [37]	S	M	S	M	S	W	Moderate
**Ryan et al., 2005** [38]	M	M	S	S	S	M	Strong
**Cistulli et al., 2023** [39]	M	M	M	N/A	S	M	Moderate
**Abdullah et al., 2018** [40]	M	M	M	N/A	M	N/A	Moderate
**Malhotra et al., 2023** [41]	M	M	W	N/A	M	N/A	Moderate
**Wojeck et al., 2023** [42]	M	M	S	S	M	W	Moderate
**Neeland et al., 2020** [43]	M	M	S	S	S	W	Moderate
**Furukawa et al., 2018** [45]	M	M	M	W	M	M	Moderate

**Tang et al., 2019** [45]	S	S	M	S	M	W	Moderate
**Sawada et al., 2018** [46]	M	M	M	W	M	W	Moderate
**Owens et al., 2021** [48]	S	S	S	S	S	S	Strong
**Pelaia et al., 2022** [49]	M	M	M	W	M	M	Moderate
**Jaffuel et al., 2021** [50]	M	S	M	W	M	S	Moderate
**Wang et al., 2023** [51]	M	M	M	W	M	M	Moderate
**Passino et al., 2021** [52]	M	M	M	W	M	S	Moderate

Abbreviations; S, strong; M, moderate; W, weak; N/A, not applicable

Table S2. Effective Public Healthcare Panacea Project - Quality Assessment Tool for Quantitative Studies Dictionary**.**

Components of EPHPP	Questions	Instructions for completion
**A. Selection Bias**	**(Q1) Are the individuals selected to participate in the study likely to be representative of the target population?**<br>1 Very likely<br>2 Somewhat likely<br>3 Not likely<br>4 Can't tell<br>**(Q2) What percentage of selected individuals agreed to participate?**<br>1 80 - 100% agreement<br>2 60 – 79% agreement<br>3 less than 60% agreement<br>4 Not applicable<br>5 Can't tell	**Strong:** The selected individuals are very likely to be representative of the target population (Q1 is 1) and there is greater than 80% participation (Q2 is 1).<br>**Moderate:** The selected individuals are at least somewhat likely to be representative of the target population (Q1 is 1 or 2); and there is 60 - 79% participation (Q2 is 2). 'Moderate' may also be assigned if Q1 is 1 or 2 and Q2 is 5 (can't tell).<br>**Weak:** The selected individuals are not likely to be representative of the target population (Q1 is 3); or there is less than 60% participation (Q2 is 3) or selection is not described (Q1 is 4); and the level of participation is not described (Q2 is 5)<br><br>**Strong - 1**<br>**Moderate - 2**<br>**Weak - 3**
**B. Study Design**	**(Q1) Indicate the study design**<br>1 Randomized controlled trial<br>2 Controlled clinical trial<br>3 Cohort analytic (two group pre + post)<br>4 Case-control	**Strong:** will be assigned to those articles that described RCTs and CCTs.<br>**Moderate**: will be assigned to those that described a cohort analytic study, a case control study, a cohort design, or an

	5 Cohort (one group pre + post (before and after))<br>6 Interrupted time series<br>7 Other specify<br>8 Can't tell<br><br>**Was the study described as randomized? If NO, go to Component C.**<br>No/Yes<br>**If Yes, was the method of randomization described?**<br>No/Yes<br>**If Yes, was the method appropriate?**<br>No/Yes	interrupted time series.<br>**Weak**: will be assigned to those that used any other method or did not state the method used.<br><br>**Strong - 1**<br>**Moderate - 2**<br>**Weak - 3**
**C. Confounders**	**(Q1) Were there important differences between groups prior to the intervention?**<br>1 Yes<br>2 No<br>3 Can't tell<br>**The following are examples of confounders:**<br>1 Race<br>2 Sex<br>3 Marital status/family<br>4 Age<br>5 SES (income or class)<br>6 Education<br>7 Health status<br>8 Pre-intervention score on outcome measure	**Strong**: will be assigned to those articles that controlled for at least 80% of relevant confounders (Q1 is 2); or (Q2 is 1).<br>**Moderate**: will be given to those studies that controlled for 60 – 79% of relevant confounders (Q1 is 1) and (Q2 is 2).<br>**Weak**: will be assigned when less than 60% of relevant confounders were controlled (Q1 is 1) and (Q2 is 3) or control of confounders was not described (Q1 is 3) and (Q2 is 4).

	**(Q2) If yes, indicate the percentage of relevant confounders that were controlled (either in the design (e.g. stratification, matching) or analysis)?**<br>1 80 – 100% (most)<br>2 60 – 79% (some)<br>3 Less than 60% (few or none)<br>4 Can't Tell	**Strong - 1**<br>**Moderate - 2**<br>**Weak - 3**
**D. Blinding**	**(Q1) Was (were) the outcome assessor(s) aware of the intervention or exposure status of participants?**<br>1 Yes<br>2 No<br>3 Can't tell<br><br>**(Q2) Were the study participants aware of the research question?**<br>1 Yes<br>2 No<br>3 Can't tell	**Strong**: The outcome assessor is not aware of the intervention status of participants (Q1 is 2); and the study participants are not aware of the research question (Q2 is 2).<br>**Moderate**: The outcome assessor is not aware of the intervention status of participants (Q1 is 2); or the study participants are not aware of the research question (Q2 is 2); or blinding is not described (Q1 is 3 and Q2 is 3).<br>**Weak**: The outcome assessor is aware of the intervention status of participants (Q1 is 1); and the study participants are aware of the research question (Q2 is 1).

		**Strong - 1**<br>**Moderate - 2**<br>**Weak - 3**
**E. Data Collection Method**	**(Q1) Were data collection tools shown to be valid?**<br>1 Yes<br>2 No<br>3 Can't tell<br><br>**(Q2) Were data collection tools shown to be reliable?**<br>1 Yes<br>2 No<br>3 Can't tell	**Strong**: The data collection tools have been shown to be valid (Q1 is 1); and the data collection tools have been shown to be reliable (Q2 is 1).<br>**Moderate**: The data collection tools have been shown to be valid (Q1 is 1); and the data collection tools have not been shown to be reliable (Q2 is 2) or reliability is not described (Q2 is 3).<br>**Weak**: The data collection tools have not been shown to be valid (Q1 is 2) or both reliability and validity are not described (Q1 is 3 and Q2 is 3).<br><br>**Strong - 1**<br>**Moderate - 2**<br>**Weak - 3**
**F. Withdrawals and Dropouts**	**(Q1) Were withdrawals and drop-outs reported in terms of numbers and/or reasons per group?**<br>1 Yes<br>2 No<br>3 Can't tell<br>4 Not Applicable (i.e. one time surveys or interviews)	**Strong**: will be assigned when the follow-up rate is 80% or greater (Q2 is 1).<br>**Moderate**: will be assigned when the follow-up rate is 60 – 79% (Q2 is 2) OR Q2 is 5 (N/A).<br>**Weak**: will be assigned when a follow-up rate is less than 60% (Q2 is 3) or if the withdrawals and drop-outs were not described (Q2 is 4).

	**(Q2) Indicate the percentage of participants completing the study. (If the percentage differs by groups, record the lowest).**<br>1 80 -100%<br>2 60 - 79%<br>3 less than 60%<br>4 Can't tell<br>5 Not Applicable (i.e. Retrospective case-control)	**Strong - 1**<br>**Moderate - 2**<br>**Weak - 3**
**Global Rating**	**GLOBAL RATING FOR THIS PAPER (circle one):**<br>1 STRONG (no WEAK ratings)<br>2 MODERATE (one WEAK rating)<br>3 WEAK (two or more WEAK ratings)<br><br>**With both reviewers discussing the ratings: Is there a discrepancy between the two reviewers with respect to the component (A-F) ratings?**<br>No/Yes<br><br>**Final decision of both reviewers (circle one):**<br>1 STRONG<br>2 MODERATE<br>3 WEAK	

Abbreviations: EPHPP, Effective Public Healthcare Panacea Project; Q1, question 1; Q2, question 2.
